# Land-based salmon aquacultures change the quality and bacterial degradation of riverine dissolved organic matter

**DOI:** 10.1038/srep43739

**Published:** 2017-03-03

**Authors:** Norbert Kamjunke, Jorge Nimptsch, Mourad Harir, Peter Herzsprung, Philippe Schmitt-Kopplin, Thomas R. Neu, Daniel Graeber, Sebastian Osorio, Jose Valenzuela, Juan Carlos Reyes, Stefan Woelfl, Norbert Hertkorn

**Affiliations:** 1 Helmholtz-Centre for Environmental Research UFZ, Department of River Ecology, Brückstraße 3a, D-39114 Magdeburg, Germany; 2 Helmholtz-Centre for Environmental Research UFZ, Department of Lake Research, Brückstraße 3a, D-39114 Magdeburg, Germany; 3 Universidad Austral de Chile, Facultad de Ciencias, Instituto de Ciencias Marinas y Limnológicas, Laboratorio de Bioensayos y Limnologia Aplicada, Casilla 567, Valdivia, Chile; 4 Helmholtz-Centre Munich, German Research Center for Environmental Health, Department of Environmental Sciences, Ingolstädter Landstraße 1, P. O. Box 1129, D-85758 Neuherberg, Germany; 5 Aarhus University, Department of Bioscience, Vejlsøvej 25, 8600 Silkeborg, Denmark

## Abstract

Aquacultures are of great economic importance worldwide but pollute pristine headwater streams, lakes, and estuaries. However, there are no in-depth studies of the consequences of aquacultures on dissolved organic matter (DOM) composition and structure. We performed a detailed molecular level characterization of aquaculture DOM quality and its bacterial degradation using four salmon aquacultures in Chile. Fluorescence measurements, ultrahigh-resolution mass spectrometry, and nuclear magnetic resonance spectroscopy of the DOM revealed specific and extensive molecular alterations caused by aquacultures. Aquacultures released large quantities of readily bioavailable metabolites (primarily carbohydrates and peptides/proteins, and lipids), causing the organic matter downstream of all the investigated aquacultures to deviate strongly from the highly processed, polydisperse and molecularly heterogeneous DOM found in pristine rivers. However, the upstream individual catchment DOM signatures remained distinguishable at the downstream sites. The benthic algal biovolume decreased and the bacterial biovolume and production increased downstream of the aquacultures, shifting stream ecosystems to a more heterotrophic state and thus impairing the ecosystem health. The bacterial DOM degradation rates explain the attenuation of aquaculture DOM within the subsequent stream reaches. This knowledge may aid the development of improved waste processing facilities and may help to define emission thresholds to protect sensitive stream ecosystems.

Streams and rivers are regarded as global hotspots of organic-matter processing and CO_2_ evasion^1^,^2^. The streambed and its biofilm microbiomes drive fundamental ecosystem processes and biogeochemical cycles^2^,^3^,^4^ through the physical fractionation and chemical processing of organic molecules. Most of the terrestrial organic carbon entering freshwater systems is either respired to CO_2_ locally or buried in sediments, and only a fraction is discharged into the ocean^4^,^5^,^6^. Inherently complex stream biofilms are hotspots of biodiversity and enzymatic and metabolic activity across all domains of life (including microalgae, bacteria, fungi, protozoans and small metazoans)^2^. Biofilms co-evolve with their respective streambed environments; land use, rather than spatial factors, such as latitude or elevation, most strongly define the community composition, diversity and capacity to perform critical ecosystem services^2^.

Many streams and rivers are affected by anthropogenic DOM loads with altered molecular composition, usually due to urban point sources^7^ or agricultural diffuse sources^8^. In Chile, pristine streams with otherwise rarely observed intact natural organic CHNO cycles^9^ occur in spatial proximity to anthropogenically affected streams. Land-based aquaculture has recently been shown to impact streams with high levels of altered DOM in northern Patagonia in Chile^10^. Chilean salmon production is economically important, contributing ~25% of the worldwide salmon yield (Chile ranks second of the world’s salmon-producing countries^11^). Salmon farming has continuously increased in recent decades; the annual salmonid production in Chile was 820,000 tons in 2012, representing a value of 4.9 billion USD (32% of the total worldwide value of salmonid production^11^). Small salmon are reared in land-based aquacultures supplied with stream water, whereas mid-sized fish are grown in cages in lakes and adult fish in cages along the coast. The effluents from land-based aquaculture pollute pristine streams with nutrients, antibiotics and organic carbon, resulting in oxygen depletion^12^ and negative consequences for the abundance and biodiversity of stream organisms, as well as for critical ecosystem functions, such as stream metabolism^13^. While aquacultures have recently started to remove suspended matter from waste water using sedimentation basins and rotating drum filters, dissolved components are still discharged untreated.

Nutrients and DOM originating from the leaching of remaining food pellets, fish faeces and fish excretions are major components released by aquacultures. One aquaculture in northern Patagonia was estimated to release DOM amounting to 21% of the carbon applied as feed and 76% of the annual fish production^10^. However, limited detailed information on the DOM composition associated with fish aquaculture is available. The DOM leached from decaying carcasses after salmon spawning has been characterized by fluorescence measurements^14^,^15^, but only two studies have applied this method to assess DOM quality from aquacultures: one study on rainbow trout in Denmark^16^ and one on salmon in Chile^10^. The latter study demonstrated that aquaculture DOM was dominated by protein-like fluorescence, which quickly degraded downstream within 2700 m^10^; however, advanced DOM specification and measurement of bacterial activity were not performed. No in-depth molecular characterization of DOM associated with aquaculture effluent has been conducted using ultrahigh-resolution Fourier transform ion cyclotron mass spectrometry (FTICR MS)^17^,^18^,^19^, nuclear magnetic resonance spectroscopy (NMR)^20^,^21^,^22^, or a combination of these techniques with excitation-emission matrices (EEM)^23^. Moreover, little is known about the consequences of aquacultures on bacterial abundance and diversity (see review^12^). An increase in bacterial number, heterotrophic activity, and extracellular enzyme activity was observed in the waters and sediments downstream of aquacultures^24^,^25^ in addition to a decline in the phosphatase activity in biofilms^26^. However, the spatial localization of the main DOM degradation in the stream, i.e., free water or benthic zone, is not clear.

In the present study, we characterized the DOM composition of pristine headwaters with low DOM concentrations and of polluted aquaculture effluents and downstream sites of four land-based aquacultures in northern Patagonia (Molco, Peuco, Huililco, and Niltre). The DOM composition was assessed using fluorescence spectroscopy, ultrahigh-resolution FTICR MS, and NMR. These measurements were complemented by the estimation of the bacterial biomass production of planktonic bacteria in stream water and epilithic biofilms, aiding in the differentiation of organic carbon processing between water and the benthic zone.

## Results

### DOM bulk characteristics

**Figure 1:**
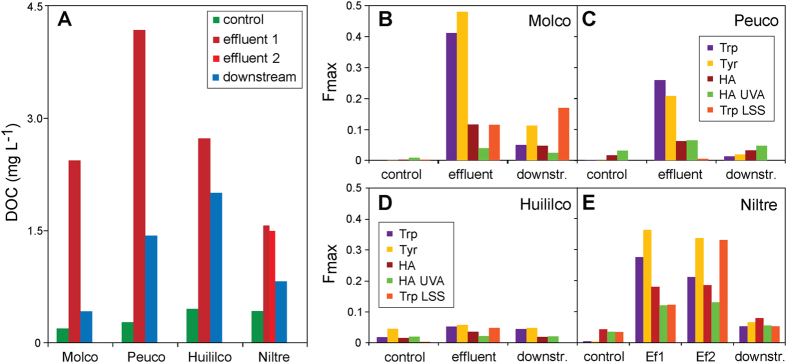
Concentrations of dissolved organic carbon (DOC) of the control sites, effluents and downstream of the aquacultures of the four sampling sites (**A**). Fluorescence intensities (F_max_ values, Raman units) of the parallel factor analysis (PARAFAC) components of the control sites, effluents and downstream of the aquacultures of the four sampling sites (**B**–**E**; Trp: tryptophan-like, Tyr: tyrosine-like, HS, HS2: humic acid-like).

The dissolved organic carbon (DOC) concentrations in the four investigated streams ranged in the order of control (0.2–0.4 mg C L^−1^) < downstream (0.4–2.2 mg C L^−1^) < effluents (1.5–4.2 mg C L^−1^) (Fig. 1A). The parallel factor analysis (PARAFAC) of the DOM fluorescence spectra identified five components (Table S1). The protein-like fluorescence of EEM was dominated by tryptophan-like (Trp/Trp2)^27^ and tyrosine-like (Tyr)^28^,^29^ components, which were shown to reflect the aquatic production of highly biodegradable DOM^30^. In addition, two distinct, humic-like fluorescence components, HS and HS2^31^, were also identified. HS, which is associated with terrestrial origins and has a relatively high molecular weight^32^,^33^, was more prevalent, whereas HS2, with microbial origin and lower molecular weight^31^,^34^, was less abundant. The pattern of the fluorescence intensities (F_max_ values) followed that of the DOC concentrations (Fig. 1). Effluents showed a particularly high fluorescence of protein-like DOM. EEM/PARAFAC analysis of aquaculture-induced DOM quality differences showed increased loadings of Trp-like and Tyr-like components by the aquacultures in the order of Molco > Niltre ~ Peuco ≫ Huililco (Fig. 1).

### Ultrahigh-resolution Fourier transform ion cyclotron (FTICR) mass spectrometry of the control sites

**Figure 2:**
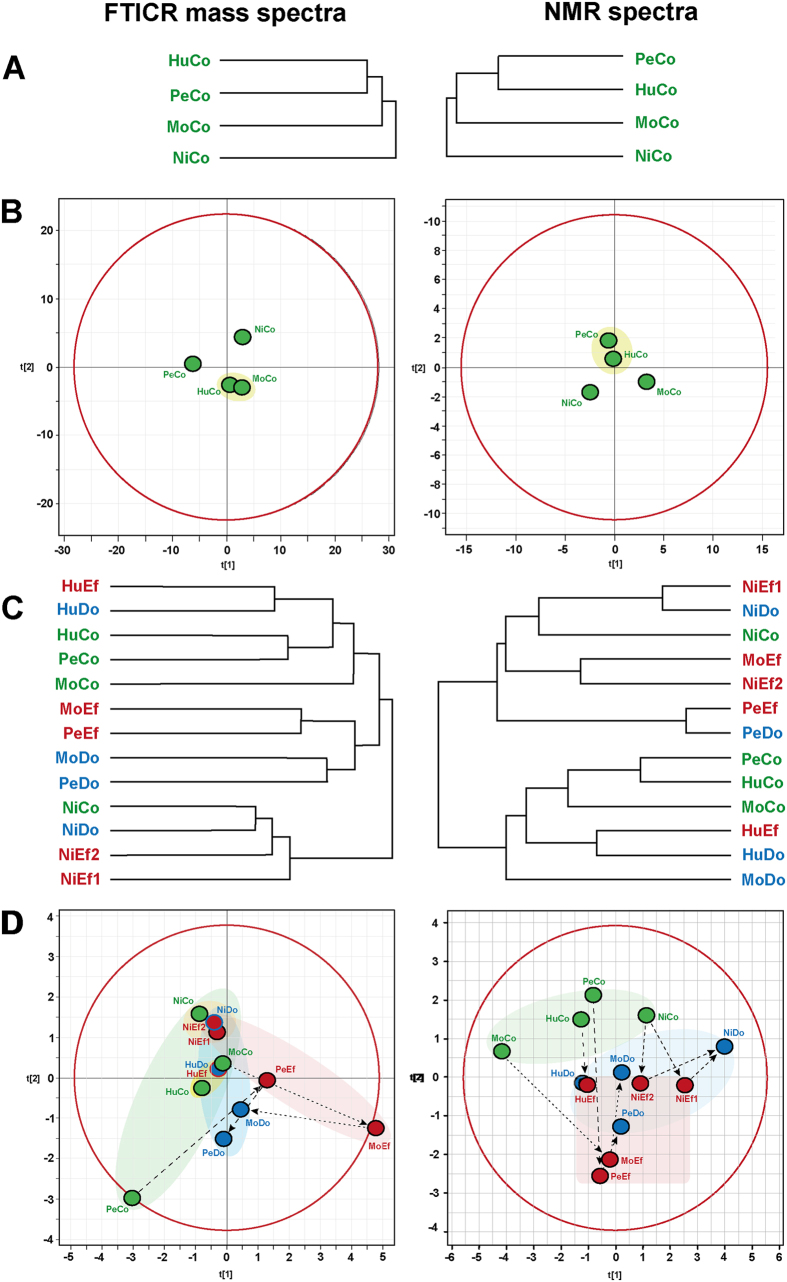
Alterations in the dissolved organic matter (DOM) characteristics as observed by (left) Fourier transform ion cyclotron mass spectrometry (FTICR MS) and (right) ^1^H nuclear magnetic resonance (NMR) spectroscopy. Top: hierarchical cluster analysis (HCA; **A**) and principal component analysis (PCA; **B**) of four pristine control DOM (cf. Figs 4A and S1). Bottom: HCA (**C**) and PCA (**D**) of the control, effluent and downstream DOM (cf. Figs S6–S9). Colour code: green, control DOM; red, aquaculture effluent DOM; blue, DOM downstream of the aquaculture.

The negative electrospray FTICR mass spectra of the DOM isolated from four pristine catchments showed broad, continual mass-peak distributions indicative of highly processed organic matter (Fig. S1; FTICR mass spectra of the control sites, van Krevelen diagrams and mass-edited H/C ratios, inter-sample ranking analysis, counts of the mass peaks, and molecular compositions common to effluents and downstream sites are shown in detail in the Supplementary Information). Overall, the mass peak distribution differed between the four streams, and the most common spacings corresponded to methylene (Δm = 14.0156 Da) and double bond equivalents (DBE: Δm = 2.1057 Da)^18^. Considerable variance was observed in the average mass at the control sites (Niltre > Peuco > Huililco > Molco) and in the relative proportions of CHNO compounds (Molco > Peuco > Huililco > Niltre), which differed from those of CHOS compounds (Molco > Peuco ≈ Huililco > Niltre; Table S2). Relative unsaturation (expressed as DBE/C) and average oxygenation (expressed as O/C ratio) were aligned (Niltre > Peuco > Huililco ≈ Molco). The van Krevelen diagrams and mass-edited H/C ratios confirmed the similarity of the control sites for Molco and Huililco DOM, as revealed by principal component analysis (PCA; Fig. 2B). FTICR MS-based inter-sample ranking analysis of CHO compounds^17^ demonstrated that the Niltre River SPE-DOM contained relatively high proportions of oxygen-rich and hydrogen-deficient (tannin-like) CHO compounds (m/z ~ 350–700) compared to all other DOM (Fig. S2). Analogous compounds were least abundant in Huililco DOM. Aliphatic components with H/C > 1.1, particularly those with m/z > 500, were less abundant in Niltre DOM than in the three other rivers. The Molco River had a greater abundance of small molecules with m/z < 500 and H/C > 1.2 than the other three streams. Overall, the large variance in the intensity ranks in all four rivers demonstrated the individuality of DOM quality in pristine waters.

### FTICR MS derived common molecules in the effluent and downstream DOM

Hierarchical cluster analysis (HCA) and PCA of the FTICR mass spectra revealed large compositional differences between pristine and effluent DOM, with intermediate positioning of downstream DOM (Fig. 2). The quality differences between the control and effluent DOM exceeded those of the effluent and downstream samples for Molco, Peuco and Huililco (Fig. 2C, S3). However, the clustering of pristine, effluent and downstream DOM was very dense for Niltre DOM and somewhat more expansive for Huililco DOM, whereas both Peuco and Molco DOM showed large scatter and differences in their trajectories (Fig. 2D). At this level of resolution, the overall chemical diversity of the control, effluent and downstream DOM differed according to catchment in the order of Niltre < Huililco < Peuco ≈ Molco. Peuco and Molco DOM showed extensive and largely different molecular alterations comparing pristine, effluent and downstream DOM (Fig. 2C,D). The parameters calculated from the mass spectra (Table S2) showed a lower average mass for effluent DOM than pristine DOM, with the exception of Huililco, which remained nearly unchanged. The intensity-weighted H/C values increased, whereas the respective O/C values decreased in the order of Molco > Peuco > Niltre > Huililco riverine DOM. All three Huililco DOM values had nearly identical bulk parameters, such as average mass, elemental ratios (H/C and O/C), and DBE/C values. The percentages of CHNO, CHOS and CHNOS compounds in effluent DOM was strongly (Molco and Peuco), moderately (Huililco) and marginally (Niltre) increased; the relative depletion of CHO compounds occurred in the order Molco > Peuco > Huililco > Niltre.

**Figure 3:**
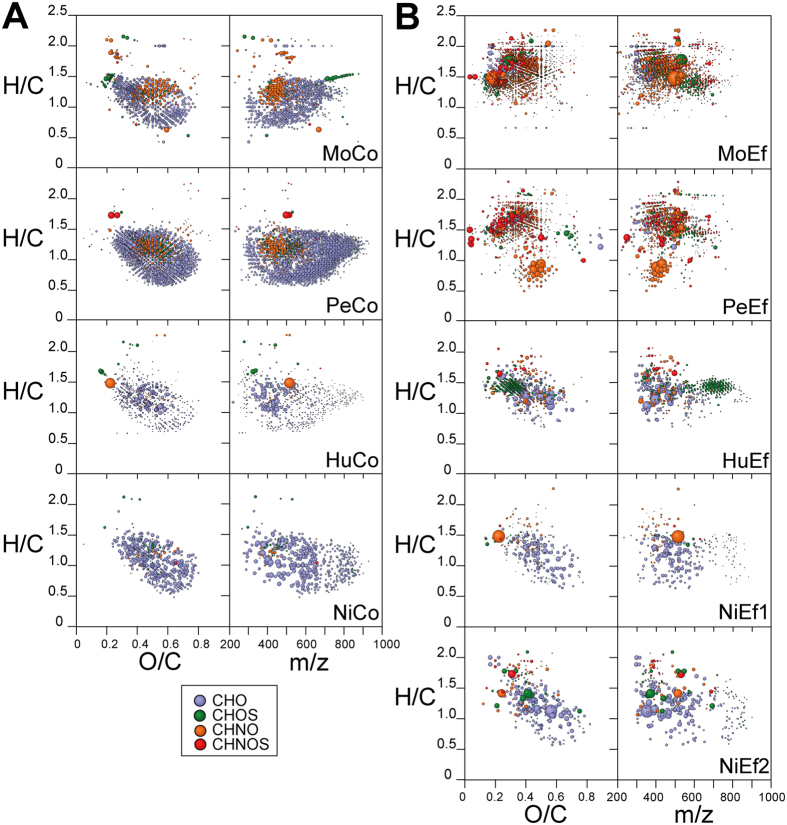
Fourier transform ion cyclotron mass spectrometry (FTICR MS) derived molecular compositions unique to (**A**) pristine and (**B**) effluent dissolved organic matter (DOM); left: van Krevelen diagrams; right: mass-edited H/C ratios. Colour code: blue, CHO; green, CHOS; orange, CHNO; and red, CHNOS molecular series. Circled area reflects the relative mass peak amplitude.

The differential analysis of pairwise FTICR mass spectra showed a clear association of particular molecular changes and the alteration of pristine to effluent DOM. Unique molecular compositions belonging to either pristine or effluent DOM showed highly individual, catchment-specific patterns in the van Krevelen diagrams and mass-edited H/C ratios (Fig. 3). The effluents of Molco and Peuco contained many saturated and oxygen-deficient CHON and CHONS (H/C > 1.2; O/C < 0.5) compounds. Newly formed compounds found in Molco and Peuco effluents also showed some admixture of low mass (m/z < 400 Da), fairly saturated (H/C > 1.4) and oxygen-deficient CHO compounds of lipid origin (Fig. 3). The effluent of Huililco contained a particular series of several dozens of CHOS compounds with intermediate unsaturation (H/C ratio 1.4 ± 0.1), relatively low oxygenation (O/C ratio: 0.3 ± 0.1) and considerable mass (m/z ~ 600–800; Fig. 3). The effluent of Huililco and both effluents of Niltre showed many high-intensity CHO compounds that were less or moderately saturated (H/C < 1.5) and a comparably low number of CHNO and CHNOS compounds.

The FTICR MS derived molecular compositions in both the effluent and downstream DOM (and absent from the control) showed distinct patterning in the van Krevelen diagrams and mass-edited H/C ratios for each of the four catchments, which was indicative of specific DOM transformations (Fig. S4). The common presence in the effluent and downstream site was characteristic for CHO/CHNO/CHNOS/CHOS compounds in the Molco River and Peuco River, with highly saturated H/C > 1.4 and O/C < 0.6 (oxygen deficient). In the Peuco River, a group of CHNO compounds with 0.5 < H/C < 1 and 0.4 < O/C < 0.6 was found in the effluent and downstream site. In the Huililco River, a suite of sulfolipids was common to the effluent and downstream site, with 1.3 < H/C < 1.7, 0.2 < O/C < 0.4 and relatively large mass (m > 600 Da). Niltre River had a near-contiguous string of carboxyl-rich alicyclic molecules (CRAM)^35^ with average H/C and O/C ratios and higher proportions of oxygenated and unsaturated CHO compounds common to the effluent and downstream site (Figs 3 and S4). In general, not all compounds present in the effluents and absent in the controls were found at the downstream sites. On a few occasions, single large-amplitude mass peaks were observed in all catchments (Table 1 and Fig. S5). This limited set of intense mass peaks, considered to be pollutants, was subjected to structure search via software freely available from the internet (www.chemspider.com), and the key lead structures were categorized.

### NMR spectroscopy of the control sites

**Figure 4:**
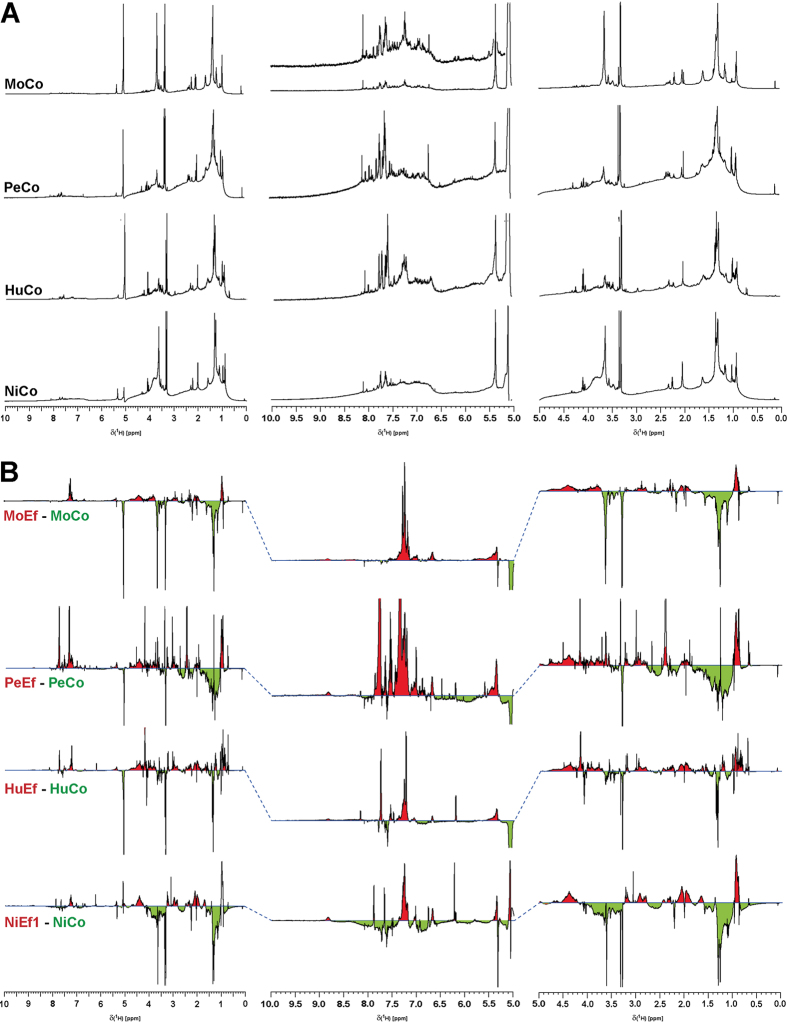
(Top): ^1^H nuclear magnetic resonance (NMR) spectra (800 MHz, CD_3_OD) of four DOM derived from “pristine” riverine catchments. (Bottom): Manual overlay difference ^1^H NMR spectra (800 MHz, CD_3_OD): effluent (red) minus pristine (green) DOM, with positive/negative amplitude referring to elevated abundance in the effluent/pristine DOM.

One-dimensional ^1^H NMR spectra of pristine riverine DOM (control) acquired in CD_3_OD showed considerable variance, demonstrating the individuality of each river catchment with respect to organic matter composition (Figs 4A and S6–S9; ^1^H NMR spectra and section integrals, ^13^C NMR spectra and section integrals, and 2D NMR spectra are shown in detail in the Supplementary Information). However, certain NMR resonance patterns were observed in all samples: the ramp-like increase of aromatic ^1^H NMR resonances from δ_H_ ~ 8.5 to 6.6 ppm indicated (poly)phenols (δ_H_ ~ 6.6–7.2 ppm); the abundant broadened NMR resonance at δ_H_ ~ 1.3 ppm indicated the presence of linear and branched aliphatics. Variable proportions of carboxyl-rich alicyclic materials (CRAM; δ_H_ ~ 1.95–3.1 ppm) and other oxygenated aliphatic compounds (**H**CO units; δ_H_ ~ 3.1–4.9 ppm) were present (Tables S3–S5), but the general NMR lineshape characteristics appeared congruent, suggesting differences in concentration rather than fundamental variance in the chemical diversity of individual pristine river catchment DOM. The control site in Molco showed major NMR aliphatic resonance at δ_H_ ~ 1.3 ppm, indicative of abundant linear and branched lipids, with secondary NMR resonances at δ_H_ ~ 2.2 (**H**Cα), 1.7 (**H**Cβ), 1.4 (**H**Cγ) and 0.9 (C**H**_3_) ppm, representing common C_3–5_ units connected to carbonyl derivatives (likely carboxylic acids: HOOC-CHα-CHβ-CHγ-C_n_-CH_3_). In contrast, Niltre showed a large proportion of methoxy groups (δ_H_ ~ 3.6–4.0 ppm), indicative of lignins, which was further corroborated by the sizable share of phenolic NMR resonances at δ_H_ ~ 6.6–7.2 ppm. Consequently, HCA separated Molco and Niltre from Peuco and Huililco, which both contained higher proportions of broad NMR resonances indicative of bulk DOM (Fig. 2). Huililco presented elevated lipid content compared with Peuco, in line with its HCA placement (Fig. 2A,B).

### NMR spectroscopy of the effluent and downstream sites

**Figure 5:**
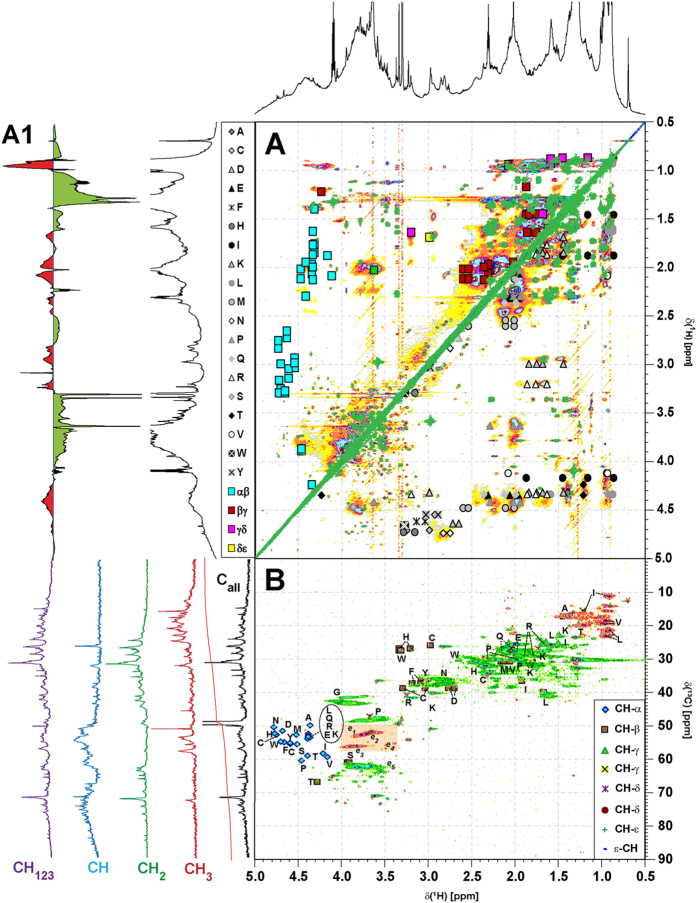
(Panel A) Overlay of ^1^H, ^1^H COSY (correlated spectroscopy; green cross peaks), ^1^H, ^1^H TOCSY (total correlated spectroscopy; coloured cross peaks) and (panel B) ^1^H, ^13^C DEPT HSQC (distortionless enhancement by polarization transfer; heteronuclear single quantum coherence) nuclear magnetic resonance (NMR) spectra (colour code: purple, CH_123_; blue, CH; green, CH_2_; red, CH_3_) of the Niltre first effluent DOM (NiEf1) aliphatic section, with cross peaks of proteinaceous amino acids (see attendant single letter code) in proteins following alanine, (**A**) annotated according to position and carbon multiplicity. (Panel A): upper left half: amino acid-derived COSY cross peaks according to positioning in peptides (blue squares); lower right half: amino acid-derived TOCSY cross peaks according to individual amino acids (individual grey symbols). (Panel B): ^1^H, ^13^C DEPT HSQC NMR spectrum; colour code: purple, CH_123_; blue, CH; green, CH_2_; red: CH_3_^57^,^58^. Orange box denotes the section of OCH_n_ cross peaks^59^ (cf. Figs 3A and S2). e_1_: aromatic methyl esters, e_2_: aliphatic methyl esters, e_3_: aromatic methyl esters, e_4_: aliphatic methyl ethers, e_5_: oxyomethylene OCH_2_, largely from carbohydrates. The abundance follows: e_5_ ≫ e_1_ ≈ e_2_: > e_3_ ≫ e_4_. Projection ^1^H NMR spectra on top (F2 dimension) represent the Niltre first effluent DOM NiEf1 (black) and difference ^1^H NMR spectra (1^st^ effluent minus pristine; NiEf1 – NiCo) Niltre first effluent DOM (cf. Fig. S9), whereas multiplicity edited ^13^C NMR subspectra (F1 dimension) are shown for panel B (cf. Fig. S10).

Difference NMR spectra [effluent minus pristine (Fig. 4B) and effluent minus downstream (data not shown)] demonstrated an increased abundance of peptide and carbohydrate metabolite signatures in the effluent DOM. ^13^C NMR spectra acquired exclusively from the Niltre River DOM indicated the prevalence of bulk organic matter signatures with variable proportions of superimposed sharp NMR resonances resulting from abundant small molecules (Fig. S10). Their contributions were marginal in the DOM from the control sites, prominent in the effluent DOM, and attenuated in the downstream DOM. ^13^C NMR section integrals revealed an increased abundance of XC**H** units in the effluent DOM, representing primarily CONHCα**H** units from peptides and proteins and OC**H** units from carbohydrates (Table S6). Common aliphatic and aromatic chemical environments showed a decreased abundance in effluent DOM (Tables S3–S5). The 2D NMR spectra showed weak cross peak patterning in pristine DOM (with the exception of the JRES NMR spectra, which favour the detection of terminal C_n_ units in mobile aliphatic chains with slow transverse NMR relaxation)^21^ and very strong patterning of abundant and intense cross peaks in the COSY, TOCSY, and HSQC NMR spectra of effluent DOM, which remained recognizable in the downstream DOM (Fig. S11). These molecular changes indicated an increased abundance of peptides (proteins) and carbohydrates, as confirmed by homonuclear and heteronuclear 2D NMR spectroscopy, in which many of the newly appearing cross peaks corresponded with those derived from random coil proteins, with a few additional lipid-derived cross peaks (Fig. 5).

### Effects on the microbial community

**Figure 6:**
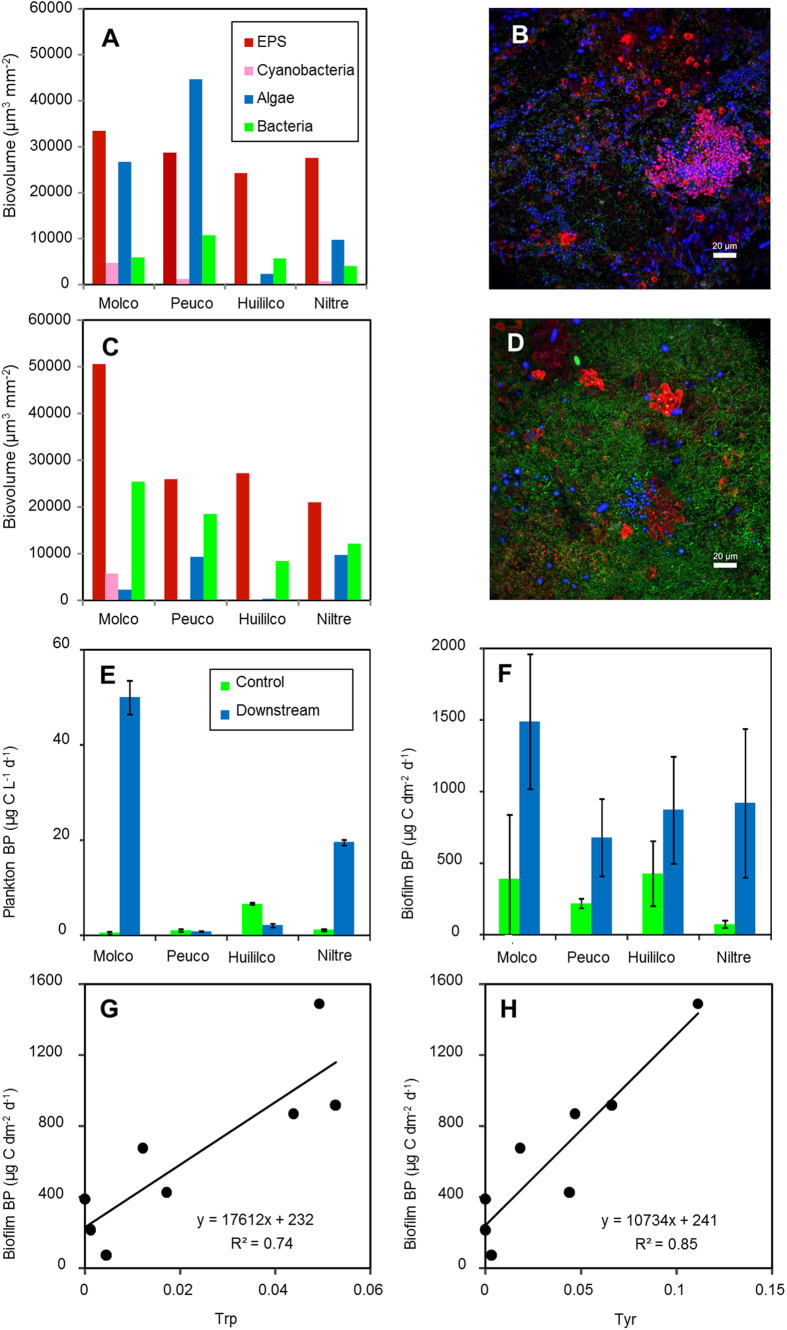
Biofilm biovolumes derived from confocal laser scanning microscopy (CLSM) image data (**A**,**C**), with two representative maximum intensity projections (**B**,**D**). (**A**,**B**) Biofilm data from a control location (Rio Niltre) showing the dominance of eukaryotic algae. (**C**,**D**) Downstream site of the aquaculture (Rio Niltre) with biofilms dominated by non-phototrophic bacteria. Colour code: blue, autofluorescence of chlorophyll *a*; purple, cyanobacteria; green, bacteria; red, lectin-specific EPS-glycoconjugates. Bacterial production of planktonic (**E**) and biofilm bacteria (**F**) at control sites and downstream of the aquacultures at the four sampling sites. Production of biofilm bacteria as a function of the fluorescence intensities (F_max_ values, Raman units) of tryptophan-like (**G**) and tyrosine-like (**H**) compounds.

Confocal laser scanning microscopy revealed that the biofilms consisted of EPS-glycoconjugates, eukaryotic algae, cyanobacteria and bacteria (Fig. 6A–D). Many algae and some cyanobacteria were observed at the control sites, whereas at the downstream sites, the gravel stones were covered by a dense bacterial biofilm. This result was confirmed by semi-quantitative biovolume data: the algae biovolumes were higher at the control sites than at the downstream sites, whereas the bacterial biovolumes were higher downstream of the effluents from the aquacultures (Fig. 6A–D). Bacterial production (BP) of planktonic bacteria was very low at Molco, Peuco and Niltre control sites (0.5–1.2 μg C L^−1^ d^−1^) and was slightly higher in Huililco (Fig. 6E,F). The planktonic BP downstream of the aquacultures followed a different pattern than the DOC concentrations: BP was either strongly increased (Molco, Niltre) or slightly (Huililco) or strongly (Peuco) decreased compared with the control. The BP of the biofilm bacteria exceeded the BP of the planktonic bacteria per dm^2^ stream area. In all streams, the BP downstream of the aquacultures was greater than the BP at the control sites upstream of the aquacultures. The BP of stream water bacteria was positively related to the F_max_ values of the Trp- and Tyr-like fluorescence components (Trp: r^2^ = 0.48, p = 0.058; Tyr: r^2^ = 0.82, p = 0.002). In addition, biofilm BP was positively correlated with the F_max_ values of the Trp- and Tyr-like compounds (Trp: r^2^ = 0.74, p = 0.006; Tyr: r^2^ = 0.85, p = 0.001; Fig. 6G,H).

## Discussion

The NMR spectra produced an unbiased depiction of the pristine riverine DOM composition and structure and showed mostly polyphenols from terrestrial input in addition to linear and branched aliphatics (possibly originating from plant waxes and other microbial lipids^36^). The individual characteristics of the NMR spectra for the control sites reflected differences in land use within the respective catchments. The Niltre River catchment is ~99% covered by natural forest (Table 2), which produces compounds with specific lignin-related features, such as abundant methoxy groups and phenols (Fig. 4A). This is in accordance with the FTICR MS-based, inter-sample ranking analyses of the CHO components^17^, which indicated higher proportions of hydrogen-deficient and oxygen-rich compounds (i.e., lignins and tannins) in the Niltre River than in the Molco, Peuco and Huililco Rivers (Fig. S2). The EEM fluorescence spectra recorded the largest loading of the humic-like component HS in the Niltre River; the lowest HS and HS2 loadings were found in the Molco River (Fig. 1).

The Molco River catchment had the lowest percentage of forest coverage (~65%) but the largest proportion of volcanic soil (~19%; Table 2). NMR and FTICR MS indicated an increased abundance of aliphatic lipids at the expense of aromatic compounds, and the EEM fluorescence spectra showed decreased loadings of humic acids. The ^1^H NMR spectra of the Molco riverine DOM showed abundant linear and branched aliphatics with depleted bulk DOM molecules, such as CRAM (Tables S3–S5 and Fig. 4A), and a distinct, narrow methoxy resonance (δ_H_ ~ 3.6 ppm), likely representing aliphatic methyl esters resulting from the quenching of reactive CHOS compounds by methanol during SPE^37^. Saturated and unsaturated sulfolipids were more abundant in the Molco DOM than in the DOM of the three other river catchments (Figs 3 and S2). FTICR MS-based inter-sample rankings analyses demonstrated low proportions of lignin (H/C > 1; O/C ~ 0.4–0.5) and tannin (H/C < 1; O/C > 0.5) components (Fig. S2A). The Peuco (which originates from a spring proximate to the aquaculture) and Huililco Rivers contained elevated proportions of bulk DOM, as demonstrated by NMR (Fig. 4A), suggesting highly processed, biogeochemical organic matter with limited contributions from individual metabolite molecules.

The FTICR mass and NMR spectra revealed alteration in DOM quality between pristine, effluent and downstream DOM: in general, the downstream DOM properties were between those of the control and effluent DOM (Fig. 2). Compared with all four pristine DOM, all four effluent DOM featured newly formed, abundant peptide/protein and carbohydrate molecules, which produced unambiguous signatures in the ^1^H spectra, composing ~35% of the overall NMR integral (Figs 4B and S6–S9), and in the ^13^C and 2D NMR spectra for the Niltre River (Figs 5 and S10, S11). These molecules remained discernible in the downstream DOM, albeit at decreased abundances (Figs S6–S10). The larger differences in the EEM/PARAFAC analysis for Molco and Niltre compared to the observations for the Peuco and Huililco Rivers reflected changes in unsaturated carbon chemical environments (Fig. 4B), in agreement with the enhanced plankton BP in Molco and Niltre in the downstream sample and the comparably smaller changes in the Peuco and Huililco Rivers.

Due to individual catchment biogeochemistry and aquaculture management practices, the molecular alterations from pristine to effluent and downstream DOM were not unidirectional: variable extents of non-conservative mixing between pristine and effluent DOM were indicated by (difference) NMR and FTICR MS spectra and HCA/PCA of the downstream DOM (Figs 2D, 3 and S3). Intrinsic catchment characteristics were retained throughout the DOM alteration and precluded the unambiguous attribution of effluent and downstream DOM from positioning in the NMR- and FTICR MS-derived HCA and PCA diagrams (Fig. 2). The abundant peptides, carbohydrates and lipids present in the effluent DOM (Fig. 5) were easily available substrates for rapid microbial consumption, as opposed to highly processed, polydisperse pristine DOM, which exhibited greater overall chemo-diversity at low concentrations of individual molecules.

The differences between the four pristine and downstream samples primarily accounted for the downstream pollution potential imposed by aquaculture. The distinct patterning of the FTICR MS-derived molecular compositions present in both effluent and downstream DOM indicated specific processing in each catchment (Fig. 2). The FTICR spectra revealed a large increase in CHNO, CHOS, and CHNOS compounds in the effluents compared to the controls in Molco and Peuco, a moderate increase in Huililco, and a minor increase in Niltre (Table S2). The DOM of both Molco and Peuco was severely impacted, with largely different chemistries in the FTICR MS spectra (Figs 3 and S4), as indicated by the divergent individual trajectories in the PCA analyses (Fig. 2D). Although variable ionization selectivity in the mass spectrometry of these complex, polydisperse mixtures^18^ could have emphasized the alteration from more conventional pristine to more heteroatom-rich effluent DOM, the ^1^H NMR spectra also indicated larger differences between pristine and effluent DOM for Peuco and Molco than for Huililco and Niltre (Figs 2D and 4B). In contrast, the effluent of Huililco showed a moderate distinction for the control site (Figs 2C and S3), suggesting a limited effect of that aquaculture due to the small effluent:control discharge ratio (Table 2). The Niltre and Huililco DOM reflected conventional biogeochemistry based on CHO compounds (Fig. 3 and S4); the Molco and in particular the Peuco Rivers showed conspicuous patterning of CHNO, CHOS, and CHNOS compounds, indicative of an anthropogenic origin of downstream DOM.

Several peaks with large amplitudes were found in the four aquacultures in both the effluent and downstream samples, and possible known chemical structures were tested by compositional matching using ChemSpider software (Table 1). Some compounds (shaded boxes) have lead structures for which xenobiotic effects have been proposed. Substances containing oxocholan structures, such as C_26_H_41_O_6_N_1_S_1_, may have an effect similar to steroids and can act as juvenoid compounds (insect hormogenic compounds, pro-drug-like agents^38^). Such substances may have phytochemical effects^39^. The C_21_H_17_O_10_N_1_ component likely incorporated pyridinedicarboxylate, which can inhibit enzymes such as the glutamate dehydrogenase found in fungi^40^. Oxazole amides, a potential substructure of the C_27_H_40_O_6_N_4,_ component, are the chemical building blocks of alkaloids^41^.

Regarding the microbial community, the low productions of planktonic bacteria at the control sites (0.5–7 μg C L^−1^ d^−1^) corresponded with the low DOC concentrations and were in the range of those measured in Amazonian streams^42^ but were near the minimum values in the Biobio River in Chile^43^ and in streams in Southern Ontario^44^. The biofilm BP in the biofilms at the control sites (71–425 μg C dm^−2^ d^−1^) corresponded to the values measured for epilithic bacterial production in small streams in Texas^45^. After calculating the areal planktonic BP using the volumetric values and mean stream depth, the biofilm bacterial production per dm^2^ was an average of 9–314 times higher than the production in stream water at the control sites and 8–175 times higher downstream of the aquacultures, indicating a clear dominance of benthic over plankton production. The abundance of bacteria in epilithic biofilms increased downstream of the aquacultures in accordance with the observed enhancement of bacterial numbers downstream of other aquacultures^24^,^25^. Moreover, we observed a decline in benthic algae and a shift towards heterotrophy^12^. A reduction in algal biomass and a shift to heterotrophic conditions was also found in periphyton downstream of a fish farm in an Andean stream^46^. The pattern of planktonic BP did not follow that of the increasing DOM concentration: some streams showed increased bacterial production, similar to that of the heterotrophic activity^24^, whereas others showed decreased BP. In contrast, the BP of biofilms increased downstream of the aquacultures in all streams, and biofilm production was positively correlated with Trp- and Tyr-like compounds (Fig. 6F–H).

Finally, we compared the observed DOM decline along a reach downstream of the aquaculture in the Molco River^10^ with the measured rates of DOM degradation (estimated from BP in the present study). A 2700-meter stretch of the Molco River, with a stream width of 5 m and a factor of two for the top and bottom surface areas of gravel stones, resulted in a total biofilm area of 27,000 m^2^. The BP in the biofilms of the Molco River amounted to 1.5 mg C dm^−2^ d^−1^ (Fig. 6F), equivalent to an average bacterial carbon demand of 6 mg C dm^−2^ d^−1^ or 25 mg C m^−2^ h^−1^ (assuming a bacterial growth efficiency of 25%)^47^,^48^. The effluent discharge (200 L s^−1^)^10^ and DOM concentration of 2 mg C L^−1^ (Fig. 1) resulted in an organic carbon load of 1.44 kg C h^−1^. The degradation of that load at a rate of 25 mg C m^−2^ h^−1^ and within two hours (the travel time for the 2700-m reach of the Molco River^10^) would necessitate a surface area of 28,800 m^2^, closely matching the computed surface area of stones (27000 m^2^). However, we measured DOM degradation during base flow conditions only. At higher discharge rates during other seasons (autumn, winter), we would expect a lower concentration of aquaculture DOM in the streams due to higher dilution and a longer distance downstream of the effluent that would be necessary for degradation due to the higher flow velocity. In addition, the lower water temperature during autumn and winter would further decrease bacterial activity.

In conclusion, highly specific molecular and biological changes were found downstream of the aquaculture operations. The DOC concentration and the fluorescence analysis of the DOM were able to distinguish pristine and polluted downstream samples. FTICR MS showed which elemental formula components could be detected as pollution in the downstream samples. The NMR analysis provided the most detailed information on the chemistry of the polluting components. To the best of our knowledge, the present study is the most detailed investigation of riverine DOM quality change due to aquaculture. The observed changes in DOM composition led to an increase in bacteria and a decrease in benthic algae downstream of the aquacultures. This shift in epilithic biofilms from autotrophy to heterotrophy alters the metabolism of the stream ecosystem, strongly impairing ecosystem health^49^. Biofilm bacterial DOM degradation was stimulated (depending on protein-like DOM compounds), which explained the disappearance of aquaculture DOM within the stream reaches. This knowledge may help to define emission thresholds for DOM to protect sensitive stream ecosystems and to design appropriate reactors for the treatment of aquaculture effluent DOM. The degradation rates measured in this study might be used to calculate the dimensions of percolated biological filters and chains of waste water treatment ponds. In light of climate change^50^, we expect that increasing temperatures will stimulate bacterial DOM degradation, but lower precipitation and discharge will decrease dilution. Thus, water quality problems will be increased due to high DOM concentrations, bacterial biomass, and oxygen demand. Finally, to build upon the description of the diversity of aquaculture DOM in the present study, further studies should investigate the effect of specific pollutants on stream ecosystems and the duration and river distance necessary for their degradation.

## Methods

### Study site and sampling

The investigations were conducted in the IX. and X. region of Chile (Region de la Araucania) in the vicinity of Lago Villarica (Fig. S12) at four aquaculture sites on the Molco (Mo), Peuco (Pe), Huililco (Hu), and Niltre (Ni; Table 2) rivers. Samples were taken 10–20 m upstream of the aquacultures (control, Co), from their effluents (Ef), and approximately 100–200 m downstream after effluent mixing (Do). Gravel stones were sampled randomly across the respective river sections. Measurements were performed during summer (between 12^th^ and 19^th^ January 2015) under summer base flow conditions (see Table 2 for discharge conditions). The river discharges were determined according to the mid-section velocity area method, for which the average velocity was measured using a WINLAB discharge velocity metre every 0.5 m along the stream width and at 0.6 of the distance from the stream surface to the streambed. The discharge was calculated as the product of the recorded velocity, depth and width of the corresponding river cross-section; the total stream discharge corresponds to the sum of each individual cross-section. There had been no rain since 1^st^ January; the maximum daily temperature on the sampling days ranged between 21 °C and 30 °C (weather station Pichoy Airport, Valdivia).

### Measurement of the DOC concentration

For the DOC analysis, water samples were transferred into acid-rinsed and combusted brown glass bottles (I-Chem 100, Merck), kept at 4 °C for a maximum of 24 h, and then filtered through 0.22-μm pore size PES membrane syringe filters (Millex Merck Millipore). DOC concentrations were measured using high-temperature catalytic oxidation (HighTOC, Elementar Systems) with a combustion temperature of 1050 °C and high-purity synthetic air (Alphagaz Airliquide) as the carrier gas, with a detection limit of 0.1 mg C L^−1^.

### Fluorescence measurement of DOM

Fluorescence measurements were conducted with a Varian Cary Eclypse fluorescence spectrometer (Santa Clara, CA, USA)^10^. Excitation from 240 to 450 nm (5 nm steps) and emission from 300 to 600 nm (2 nm steps), with a slit width of 5 nm, were measured to produce excitation-emission matrices (EEM)^28^. All samples were measured at room temperature and were corrected to the absorbance spectra recorded in the range of 190 to 800 nm for the instrument baseline. Absorbance was measured in 1-cm cuvettes using a Pharo Spectroquant 200 spectrophotometer for the inner-filter correction of the fluorescence measurements (Darmstadt, Germany). Daily measurements of the area under the Raman peak of MilliQ water were recorded to assess instrument stability^51^. Primary and secondary inner-filter effects were removed using mathematical inner-filter corrections^52^. Excitation corrections were normalized by the area under the Raman peak at the 350 nm excitation wavelength^51^. These corrections were conducted using the FDOMcorr toolbox^53^ for Matlab (version R2012a, MathWorks, Ismaning, Germany) and allowed the best possible comparability to other DOM fluorescence studies^51^,^53^.

### High-field FTICR mass spectrometry of SPE-DOM

For DOM enrichment, 10 L of stream water was filtered through GFF and acidified with HCl to pH 2.0–2.5. Bond Elut SPE PPL cartridges (1 g; Agilent Technologies) were rinsed with 5 mL of 100% methanol, 5 mL of pure water and 5 mL of 0.01 N HCl. Pre-filtered stream water (8 L) was filtered through each cartridge. After rinsing with 10 mL of 0.01 N HCl, drying, and eluting with 10 mL of 100% methanol (LCMS grade), samples were stored at −25 °C. High-field Fourier transform ion cyclotron (FTICR) mass spectra were acquired using a 12 Tesla Bruker Solarix mass spectrometer (Bruker Daltonics, Bremen, Germany) hyphenated to an Apollo II electrospray ionization source in negative mode [ESI(−)]^23^. The SPE-DOM samples were injected into the electrospray source using a microliter pump at a flow rate of 120 μL h^−1^, a nebulizer gas pressure of 138 kPa, and a drying gas pressure of 103 kPa. A source heater temperature of 200 °C was maintained to ensure rapid desolvation in the ionized droplets. The spectra were acquired with a time domain of four megawords in [ESI(−)], and 500 scans were accumulated for each mass spectrum. All spectra were internally calibrated using appropriate reference mass lists. Data processing was conducted using Compass Data Analysis 4.0 (Bruker, Bremen, Germany). Possible elemental formulas were assigned using our own software (a formula calculator). The generated formulas were validated by setting sensible chemical constraints [signal-to-noise (S/N) > 3, N rule, O/C ratio ≤ 1, H/C ratio ≤ 2n + 2, element counts: C ≤ 100, H ≤ 200, O ≤ 80, N ≤ 6, S ≤ 3 and mass accuracy window (set at ± 500 ppb)], and the final molecular formula assignments were branded into groups containing CHO, CHNO, CHOS or CHNOS molecular compositions, which were used to reconstruct the group-selective mass spectra. The FTICR MS and NMR datasets were processed using Hierarchical Clustering Explore 3.5 after normalizing the data using unit variance scaling. Samples and variables were then clustered by applying average linkage (UPGMA) and Pearson correlation coefficient to analyse the distance measure. SIMCA-P version 9.0 from UNIMETRICS was used for the PCA plots.

### NMR spectroscopy of SPE-DOM

Proton-detected NMR spectra of methanolic riverine SPE-DOM extracts were acquired using a Bruker Avance III NMR spectrometer at 800.13 MHz (B_0_ = 18.7 T) and 283 K from ~3 to 7 mg (cf. Table S7) of solid SPE-DOM obtained by evaporation of the original methanol-h_4_ solution. Proton NMR spectra were acquired in approximately 170 μL of CD_3_OD (Merck. 99.95% ^2^H) solution with a 5-mm z-gradient ^1^H/^13^C/^15^N/^31^P QCI cryogenic probe (90° excitation pulses: ^13^C ~ ^1^H ~ 10 μs) in sealed 3.0-mm Bruker MATCH tubes. 1D ^1^H NMR spectra were recorded with a spin-echo sequence (10 μs delay) to allow for high-Q probe ringdown, and classical pre-saturation to attenuate the residual water present “*noesypr1d*”, typically using 512 scans (5 s acquisition time, 5 s relaxation delay, 1 ms mixing time; 1 Hz exponential line broadening). ^13^C NMR spectra were acquired with a Bruker Avance III NMR spectrometer at 500.13 MHz (B_0_ = 11.7 T) at 283 K with a 5-mm z-gradient ^1^H/^1^H/dual cryogenic probe (90° excitation pulses: ^13^C ~ 10 μs; ^1^H ~ 16.5 μs). A Bray-Curtis similarity assessment was performed by cluster analysis of the 800 MHz spectra in the chemical shift range δ_H_ = 0.7–8.7 ppm, with the exclusion of the methanol (δ_H_ = 3.2–3.4 ppm) and residual water (δ_H_ = 4.7–5.2 ppm) NMR resonances by means of AMIX-based bucket analysis (0.001 ppm uniform width, normalized total ^1^H NMR integral = 100%).

The one-bond coupling constant ^1^J(CH) used in the 2D ^1^H,^13^C DEPT HSQC spectra (*hsqcedetgpsisp2.2*) was set to 145 Hz; other conditions: ^13^C 90 deg decoupling pulse, GARP (70 μs); 50 kHz WURST 180 degree ^13^C inversion pulse (Wideband, Uniform, Rate, and Smooth Truncation; 1.2 ms); F2 (^1^H): spectral width of 5981 Hz (11.96 ppm); 1.25 s relaxation delay; F1 (^13^C): SW = 17,607 Hz (140 ppm). HSQC-derived NMR spectra were computed to an 8192 × 1024 matrix. The absolute value JRES, phase-sensitive COSY and echo-antiecho TOCSY spectra (with solvent suppression: *jresgpprqf, cosygpph19, dipsi2etgpsi19*) used a spectral width of 5498 Hz [JRES (F1) = 50 Hz] and were computed to a 16384 × 2048 matrix [JRES/TOCSY (F1) = 128/4096]. The other NMR acquisition conditions are given in Table S7.

### Confocal laser scanning microscopy (CLSM)

Structural analysis of the microbial biofilm community was conducted by CLSM using a TCS SP5 X (Leica)^54^. Extracellular polymeric substances (EPS) were stained by the lectin AAL (*Aleuria aurantia*) (Vector Laboratories) conjugated with the fluorochrome Alexa568 (Molecular Probes). Bacteria were stained with the nucleic acid specific fluorochrome Syto9 (Molecular Probes). Excitation was performed at 500 nm (reflection, Syto9), 578 nm (AAL-Alexa568, cyanobacterial autofluorescence) and 633 nm (cyanobacterial and algal autofluorescence). Emission signals were collected sequentially for reflection (495–505 nm), Syto9 (515–560 nm) and chlorophyll A (650–720 nm) in one scan. The emission of AAL-Alexa568 was recorded in a second scan. Three gravel stones per site were used for imaging, and three images were recorded for each, from the top and bottom sides. The digital signals for bacteria, EPS-glycoconjugates, cyanobacteria and chlorophyll autofluorescence were extracted using JImageAnalyser^55^ software. Semi-quantitative biovolume values were estimated after manual thresholding. The EPS-glycoconjugates and cyanobacteria data, as well as the cyanobacteria and algae present in two channels, were separated using the Imaris ver. 7.7.2 (Bitplane) co-localization tool.

### Bacterial biomass production

The production of pelagic bacteria in the stream water and of biofilm bacteria on the gravel stones was measured using the leucine technique^54^,^56^. For free-water bacteria, triplicate 5 mL aliquots and one formalin-treated control (3.7%, final concentration) were spiked with ^14^C-leucine (10.8 MBq mmol^−1^, Sigma, 50 nM final concentration). Samples were incubated *in situ* within the stream for 1 h in the dark. Incorporation was stopped using formalin, and 0.6 mL of 50% trichloroacetic acid (TCA) was added. Proteins were extracted for 15 min and filtered through 0.2-μm Nuclepore membranes. Filters were rinsed twice with 1 mL of 5% TCA and once with 80% ethanol. After dissolving the filters in 0.5 mL of Soluene (Packard) and adding 2.5 mL of biodegradable counting scintillant (Amersham) to each scintillation vial, radioactivity was measured using a liquid scintillation analyser (LS 6500, Beckman). The external standard ratio method was used for quenching, and bacterial carbon production was calculated^56^. The production of biofilm bacteria was also estimated based on leucine incorporation. Gravel stones of approximately 1 cm in length were transferred to scintillation vials and covered with 4 mL of sterile-filtered stream water. Triplicate aliquots and one formalin-treated control (3.7%, final concentration) were spiked with ^14^C-leucine (5 mM final concentration). After *in situ* incubation for 1 h and extraction with TCA on ice, the biofilms were removed from the stones by ultrasonication for 1 min (20 kHz, 20%; vibra cell VCX 130, Sonics, USA). Stones were removed and rinsed, and the supernatant was filtered and measured as described above. To estimate the surface area of the rocks, they were wrapped in tin foil, and the weight of the foil was related to the weight of one cm^2^ foil.

## Additional Information

**How to cite this article**: Kamjunke, N. *et al*. Land-based salmon aquacultures change the quality and bacterial degradation of riverine dissolved organic matter. *Sci. Rep.* **7**, 43739; doi: 10.1038/srep43739 (2017).

**Publisher's note:** Springer Nature remains neutral with regard to jurisdictional claims in published maps and institutional affiliations.

## Supplementary Material

Supplementary Information

Supplementary Dataset 1

## Figures and Tables

**Table 1:** 

Entry	Location	Formula	Mass	Monoisotopic mass	Number of results	Type of lead structures
1	Huililco	C_17_H_28_O_3_S_1_	312.4674	312.175903	68	lipid
2	Huililco	C_27_H_40_O_5_N_4_	500.6303	500.299866	137	*N-heterocyclic, non-natural product*
3	Huililco	C_18_H_22_O_10_	398.3613	398.121307	32	glycosylated aromatic natural product
4	Huililco	C_16_H_18_O_9_	354.3087	354.095093	73	glucosylated cinnamic acid
5	Huililco	C_22_H_30_O_8_	422.4688	422.194061	112	polyether
6	Molco	C_26_H_45_O_6_N_1_S_1_	499.7036	499.296753	51	*oxocholan amide sulfonic acid*
7	Molco	C_27_H_40_O_6_N_4_	516.6297	516.29480	75	*oxazol amide, pyridinetrione*
8	Molco	C_27_H_48_O_8_S_1_	532.7302	532.307007	26	*oxocholan sulfonic acid*
9	Molco	C_26_H_45_O_8_N_1_S_1_	531.7024	531.28656	4	*oxocholan amide sulfonic acid*
10	Molco	C_22_H_34_O_5_	378.5024	378.240631	346	cholan, decaline
11	Peuco	C_27_H_40_O_5_N_4_	500.6303	500.299866	137	*N-heterocyclic, non-natural product*
12	Peuco	C_21_H_17_O_10_N_1_	443.3604	443.085236	5	*2,6-pyridinedicarboxylate*
13	Peuco	C_20_H_17_O_10_N_1_	431.3497	431.085236	5	natural product, polyester
14	Peuco	C_26_H_41_O_6_N_1_S_1_	495.6718	495.265459	6	*macrocycle, oxocholane amide sulfonic acid*
15	Peuco	C_23_H_31_O_1_N_1_S_3_	433.6933	433.156780	0	new compound
16	Niltre	C_26_H_45_O_6_N_1_S_1_	499.7036	499.296753	51	*oxocholane amide sulfonic acid*

Noticeable mass peaks with large amplitudes, characteristic of the respective effluent SPE-DOM, annotated by means of compositional match using ChemSpider software (www.chemspider.com). The main lead structures are indicated, suggesting anthropogenic origins for many of these compounds (suspected xenobiotic compounds of anthropogenic origin are printed in italic font).

**Table 2:** 

Stream	Rio Molco	Rio Peuco	Rio Huililco	Rio Niltre
Location	Molco Alto	Melipeuco	Catripulli	Niltre (Panguipulli)
S	39°20´07.4”	38°50´32.8”	39°22'50.04”	39°42'27.94”
W	72°05´38.6”	71°40´10.6”	71°41'20.92”	72°12'25.65”
Q_cont_ (m^3^ s^-1^)	0.65	0.59	19.5*	0.14
Q_effl_ (m^3^ s^−1^)	0.44	0.85	2.5	0.15
Fish biomass (t)	50	36**	150	17
Length (km)	15	─	18	9
Area (km^2^)	14	─	64	22
Forest (%)	65	─	78	99
Bush and grass (%)	10	─	13	0
Volcanic (%)	19	─	5	0

Upper part: Location, discharge rate (Q) and fish biomass (value provided by aquaculture companies) of the sampling sites. Lower part: Size and most important land use forms within the catchments (stream length, catchment area, native forest, bush forest, grass land, volcanic soil). *Computed from Q_effl_ and the conductivities of the control, effluent and downstream sites. **Estimated from an annual production of 331 t using the biomass/production ratio of the Molco River. ─Rio Peuco has no catchment as its source is immediately upstream of the aquaculture.
