# Supplementary material for: Land-based salmon aquacultures change the quality and bacterial degradation of riverine dissolved organic matter
Source: Sci Rep. 2017 Mar 3;7:43739. doi: 10.1038/srep43739 (PMC5335611; doi:10.1038/srep43739)
Supplement: Supplementary Information [file srep43739-s1.pdf]

Land-based salmon aquacultures change the quality and bacterial degradation of riverine dissolved organic matter

Supplementary Information

Norbert Kamjunke[1,2]*, Jorge Nimptsch[3], Mourad Harir[4], Peter Herzsprung[2], Philippe Schmitt-Kopplin[4], Thomas R. Neu[1], Daniel Graeber[5], Sebastian Osorio[3], Jose Valenzuela[3], Juan Carlos Reyes[3], Stefan Woelfl[3], Norbert Hertkorn[4]

[1]Helmholtz-Centre for Environmental Research UFZ, Department of River Ecology, Brückstraße 3a, D-39114 Magdeburg, Germany

[2]Helmholtz-Centre for Environmental Research UFZ, Department of Lake Research, Brückstraße 3a, D-39114 Magdeburg, Germany

[3]Universidad Austral de Chile, Facultad de Ciencias, Instituto de Ciencias Marinas y Limnológicas, Laboratorio de Bioensayos y Limnologia Aplicada, Casilla 567, Valdivia, Chile

[4]Helmholtz-Centre Munich, German Research Center for Environmental Health, Department of Environmental Sciences, Ingolstädter Landstraße 1, P. O. Box 1129, D-85758 Neuherberg, Germany

[5]Aarhus University, Department of Bioscience, Vejlsøvej 25, 8600 Silkeborg, Denmark.

*corresponding author: Tel.: +49 391 8109434, fax: +49 391 8109150,
e-mail: norbert.kamjunke@ufz.de

Running head: DOM from salmon aquacultures

Key words: aquaculture, bacterial production, DOM, EEM, FT-ICR MS, NMR, Confocal Laser Scanning Microscopy (CLSM)

Supplementary results

Table S1. Results of Open Fluor data base query (www.openfluor.org)[1]. Minimum similarity score 0.95.

component	excitation & emission maximum range	references
C1 (Tryptophane like) (Trp)	Ex 275, Em 346.5	C1[2]<br>C5[3]<br>C7[4]
C2 (Tyrosin like) (Tyr)	Ex 275, Em 309	C1[5]<br>C3[6]<br>C3[7]<br>C4[8]<br>C7[9]
C3 (Humic acid like) (HS)	Ex 240, Em 419	C1[8]<br>C1[9]<br>C1[10]<br>C1[11]<br>C1[12]<br>C1[13]<br>C2[6]<br>C2[14]<br>C3[15]<br>C3[16]<br>C6[4]
C4 (Humic acid like) (HS2)	Ex 240, Em 483.5	C1[6]<br>C1[14]<br>C1[17]<br>C2[1]<br>C2[4]<br>C2[16]<br>C2[18]<br>C2[19]<br>C3[20]<br>C3[21]<br>C3[22]
C5 (Tryptophane) (Trp2)	Ex 240, Em 337	C4[10]<br>C5[3]<br>C5[6]<br>C5[7]<br>C7[4]<br>C7[22]

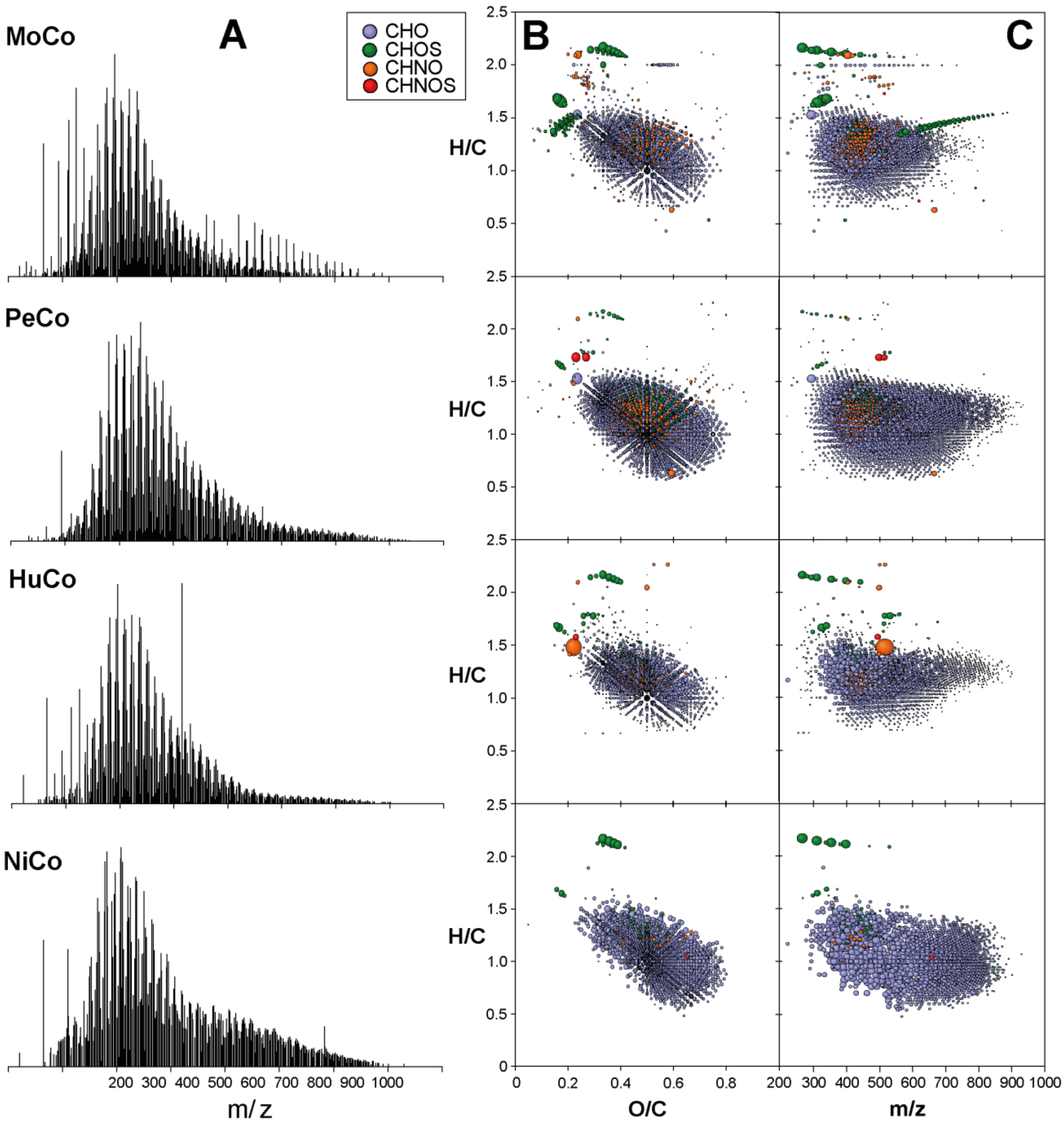

Fig. S1. Negative ESI FTICR mass spectra of DOM isolated from four "pristine" riverine catchments, with (A) mass spectra, (B) van Krevelen diagrams and (C) mass-edited H/C ratios. Color code, blue: CHO; green: CHOS; orange: CHNO, and red: CHNOS molecular series.

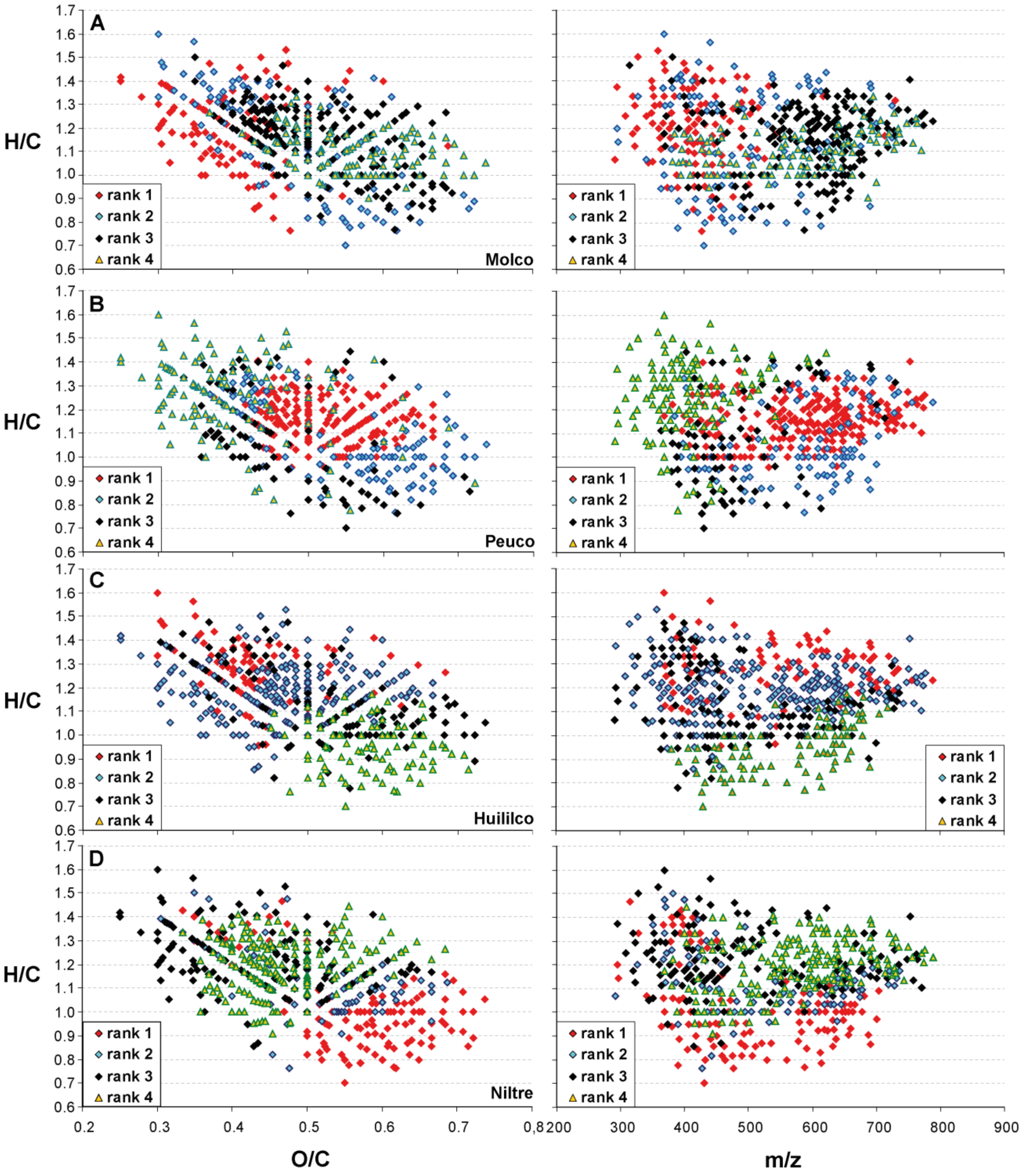

Fig. S2. Inter-sample ranking analysis of DOM from four pristine rivers (CHO compounds only); relative abundance is in the order rank 1 > rank 2 > rank 3 > rank 4[23].

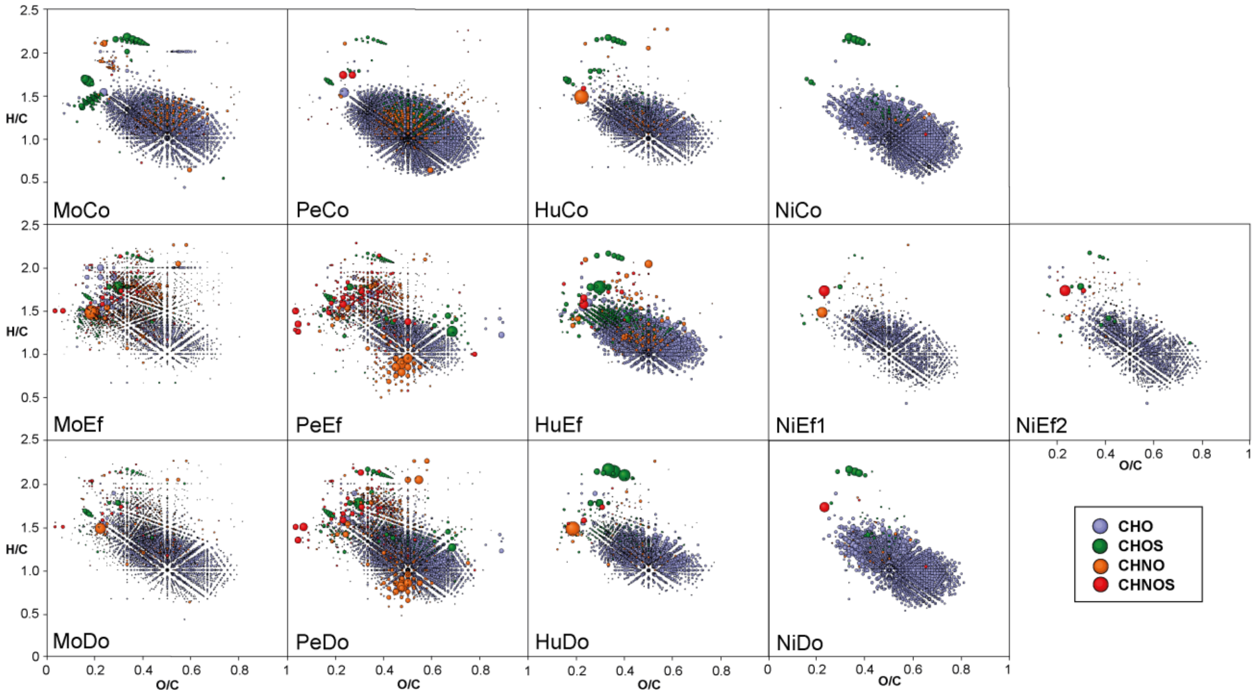

Fig. S3. Van Krevelen diagrams derived from negative ESI FTICR mass spectra of DOM from four catchments; Mo: Molco, Pe: Peuco, Hu: Huililco, Ni: Niltre river; Co: control (pristine), Ef: effluent, Do: downstream DOM. Color code: blue: CHO, (green) CHOS, (orange) CHNO and (red) CHNOS molecular series.

Table S2. Counts of mass peaks as computed from negative electrospray (ESI) 12 T FTICR mass spectra for singly charged ions, shown for control, effluent, and downstream DOM. Shaded boxes refer to intensity weighted values.

**river**	**Molco**			**Peuco**		
**SPE-DOM**	**MoCo**	**MoEf**	**MoDo**	**PeCo**	**PeEf**	**PeDo**
**CHO components**	1348	1015	1751	2369	1057	1939
**CHOS components**	169	564	331	232	298	374
**CHON components**	196	645	440	240	354	473
**CHNOS components**	21	554	194	25	321	218
**total number of mass peaks**	1734	2778	2716	2866	2030	3004
**% CHO components**	77.7	36.5	64.5	82.7	52.1	64.6
**% CHOS components**	9.8	20.3	12.2	8.1	14.7	12.5
**% CHNO components**	11.3	23.2	16.2	8.37	17.4	15.8
**% CHNOS components**	1.2	19.9	7.1	0.9	15.8	7.3
**average H [%]**	46.2	51.6	47.8	43.7	47.5	45.9
**average C [%]**	37.0	34.3	36.0	37.6	35.8	36.5
**average O [%]**	16.5	12.0	15.3	18.6	15.1	16.9
**average N [%]**	0.12	1.66	0.58	0.06	1.07	0.48
**average S [%]**	0.13	0.50	0.29	0.05	0.49	0.23
**average H/C**	1.25	1.5	1.33	1.16	1.33	1.26
**average O/C**	0.45	0.35	0.43	0.5	0.42	0.46
**average C/N**	326	21	63	603	33	75
**average C/S**	269	67.8	126	906	73.2	165
**DBE average**	9.44	6.98	8.68	11.14	8.72	9.53
**DBE/C average**	0.42	0.32	0.39	0.46	0.4	0.42
**mass weighted average [Da]**	458.5	442.8	462	510.2	459.8	474.1

**river**	**Huililco**			**Nilte**			
**SPE-DOM**	**HuCo**	**HuEf**	**HuDo**	**NiCo**	**NiEf1**	**NiEf2**	**NiDo**
**CHO components**	1440	1084	1061	1458	1061	1135	1154
**CHOS components**	138	320	265	52	53	67	45
**CHON components**	92	114	94	20	48	35	23
**CHNOS components**	6	27	21	2	15	22	6
**total number of mass peaks**	1676	1545	1441	1532	1177	1259	1228
**% CHO components**	85.9	70.2	73.6	95.2	90.1	90.2	94.0
**% CHOS components**	8.2	20.7	18.4	3.4	4.5	5.3	3.7
**% CHNO components**	5.5	7.4	6.5	1.3	4.1	2.8	1.9
**% CHNOS components**	0.4	1.8	1.5	0.1	1.3	1.8	0.5
**average H [%]**	45.5	46.2	46.6	41.7	43.8	43.0	41.8
**average C [%]**	37.1	36.9	36.6	38.1	37.4	37.6	38.0
**average O [%]**	17.2	16.7	16.4	20.1	18.5	19.1	20.1
**average N [%]**	0.14	0.11	0.22	0.02	0.25	0.11	0.03
**average S [%]**	0.08	0.14	0.19	0.03	0.06	0.12	0.05
**average H/C**	1.23	1.25	1.27	1.09	1.17	1.15	1.1
**average O/C**	0.46	0.45	0.45	0.53	0.49	0.51	0.53
**average C/N**	245	310	169	3161	145	333	1336
**average C/S**	469	253	192	1075	532	308	827
**DBE average**	9.99	9.71	9.44	12.29	10.97	11.42	11.86
**DBE/C average**	0.43	0.42	0.41	0.49	0.46	0.46	0.48
**mass weighted average [Da]**	479.5	478.2	476.4	536	504.6	520.2	521

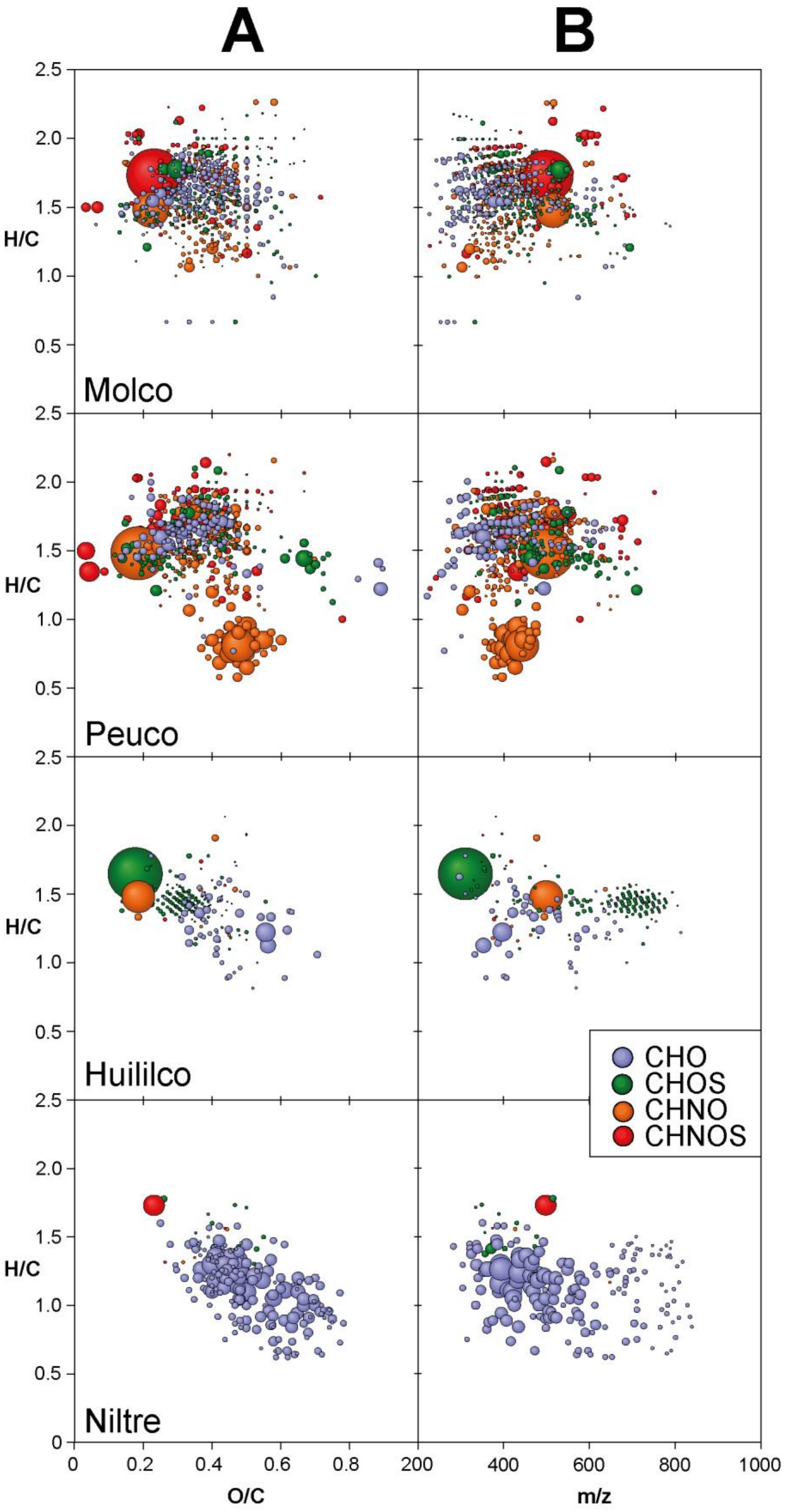

Fig. S4. Molecular compositions, common to effluent and downstream site SPE-DOM from four catchments; (A) van Krevelen diagrams and (B) mass-edited H/C ratios. The downstream site values were used as a basis for visualization of intensities.

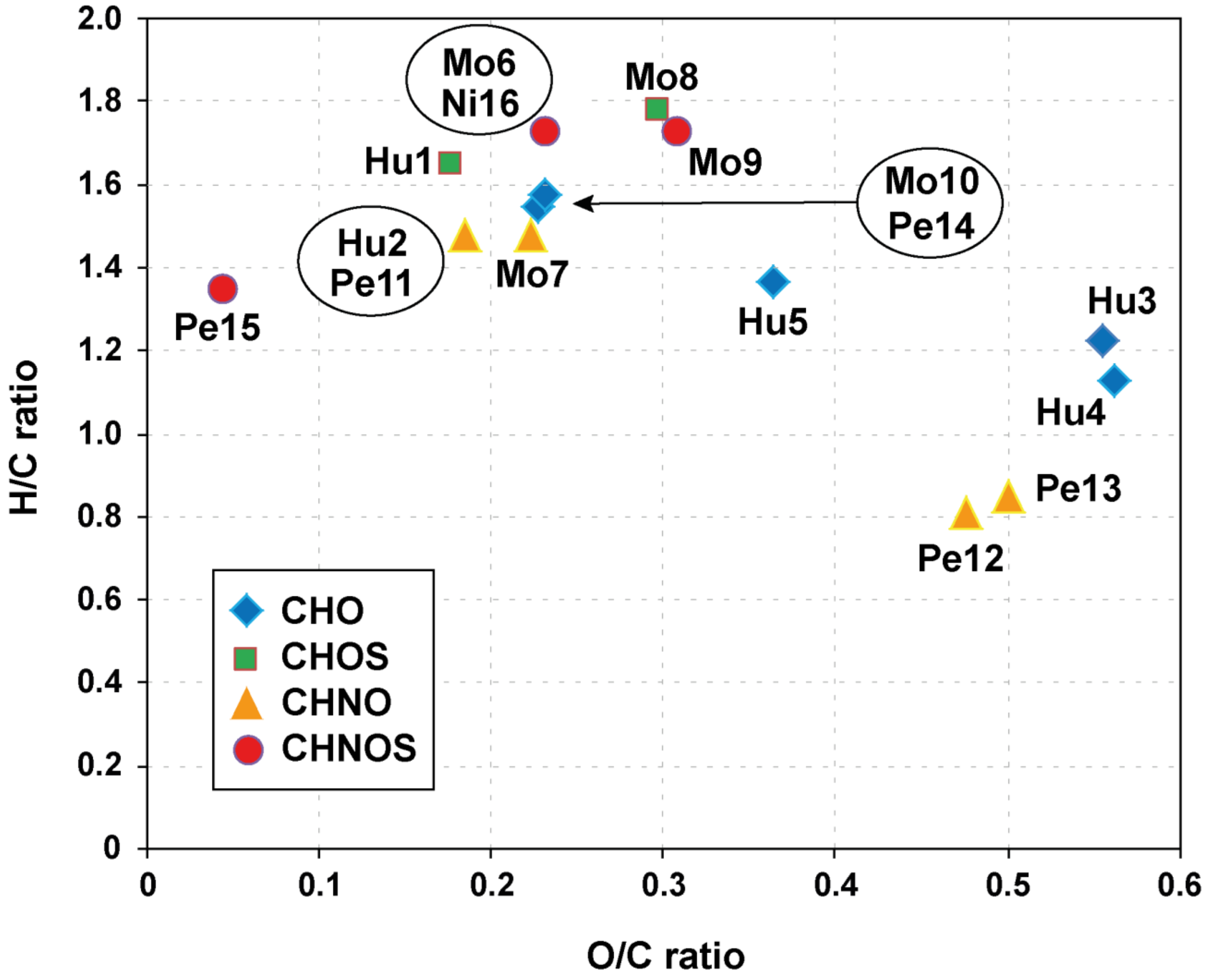

Fig. S5. FTICR MS derived van Krevelen diagrams of main polluting components in effluent SPE-DOM according to molecular series; annotation: cf. Table 1.

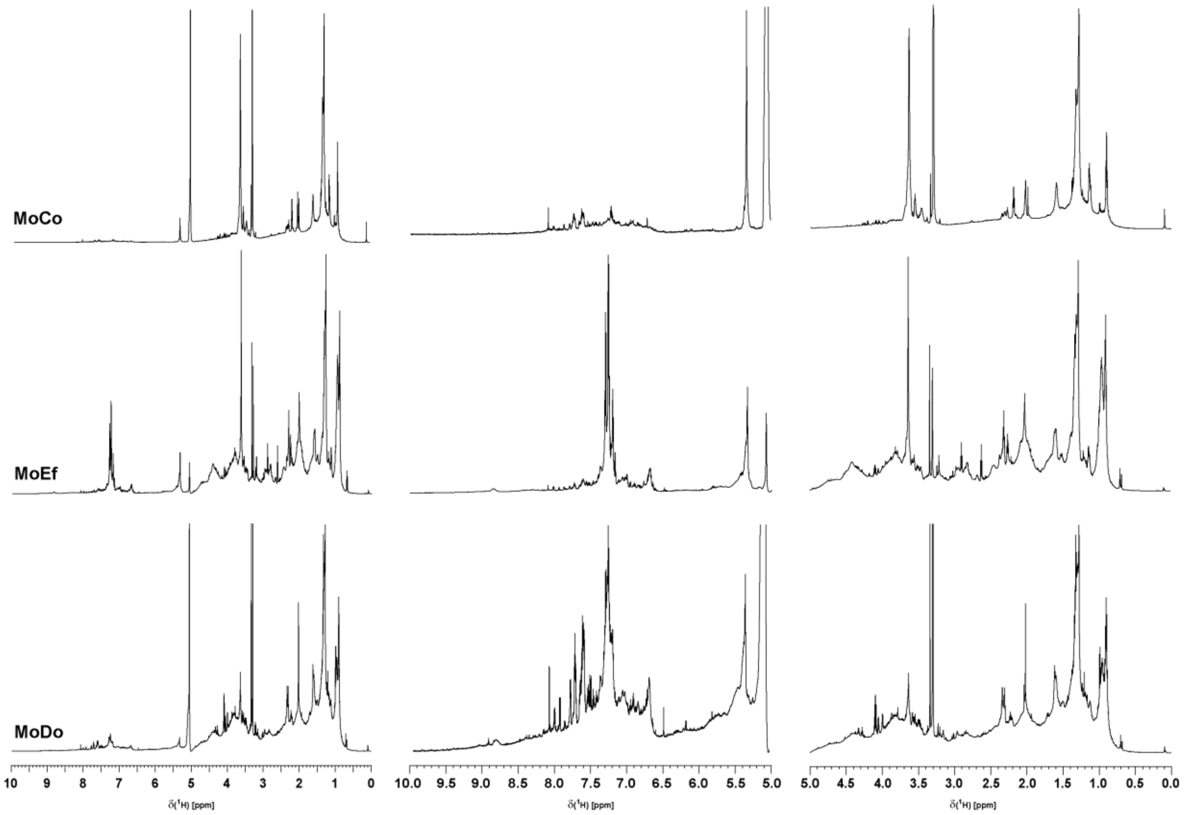

Fig. S6. ^{1}H NMR spectra (800 MHz, CD_3OD) of Molco riverine DOM; top: pristine, control (MoCo), center: effluent (MoEf), bottom: downstream (MoDo).

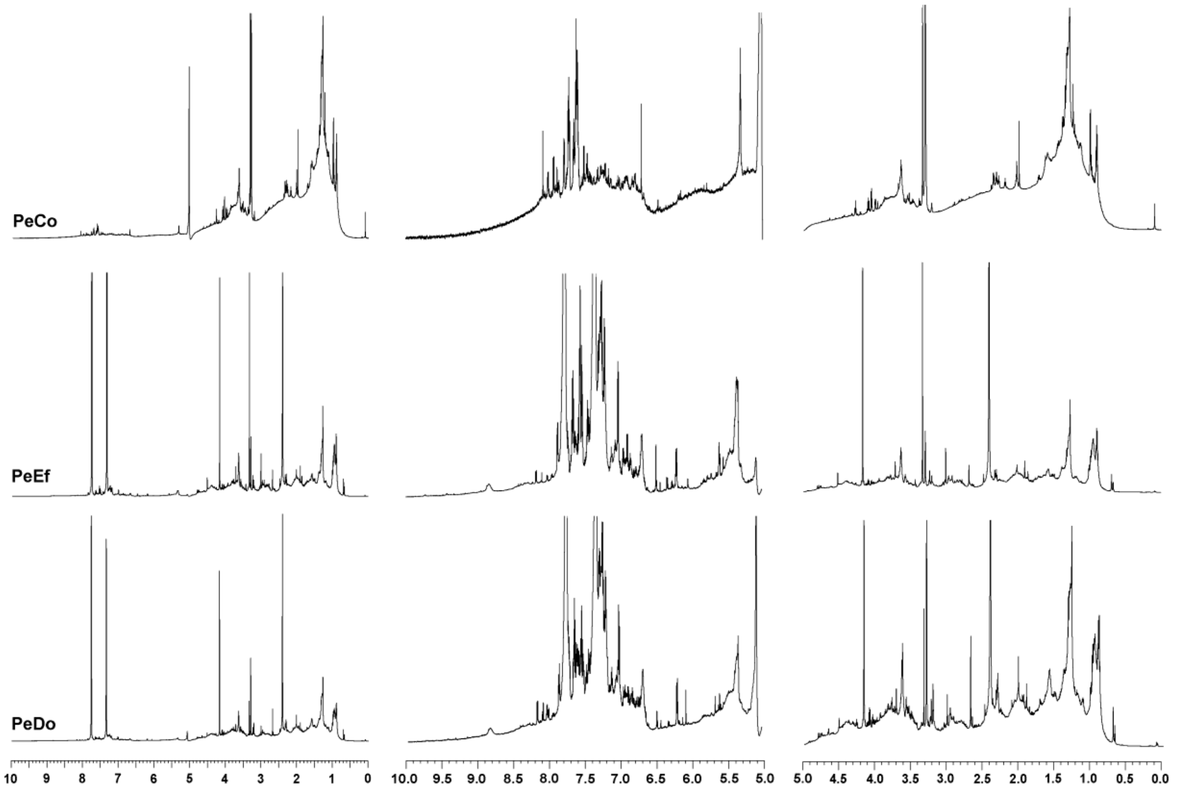

Fig. S7. ^{1}H NMR spectra (800 MHz, CD_3OD) of Peuco riverine DOM; top: pristine, control (PeCo), center: effluent (PeEf), bottom: downstream (PeDo).

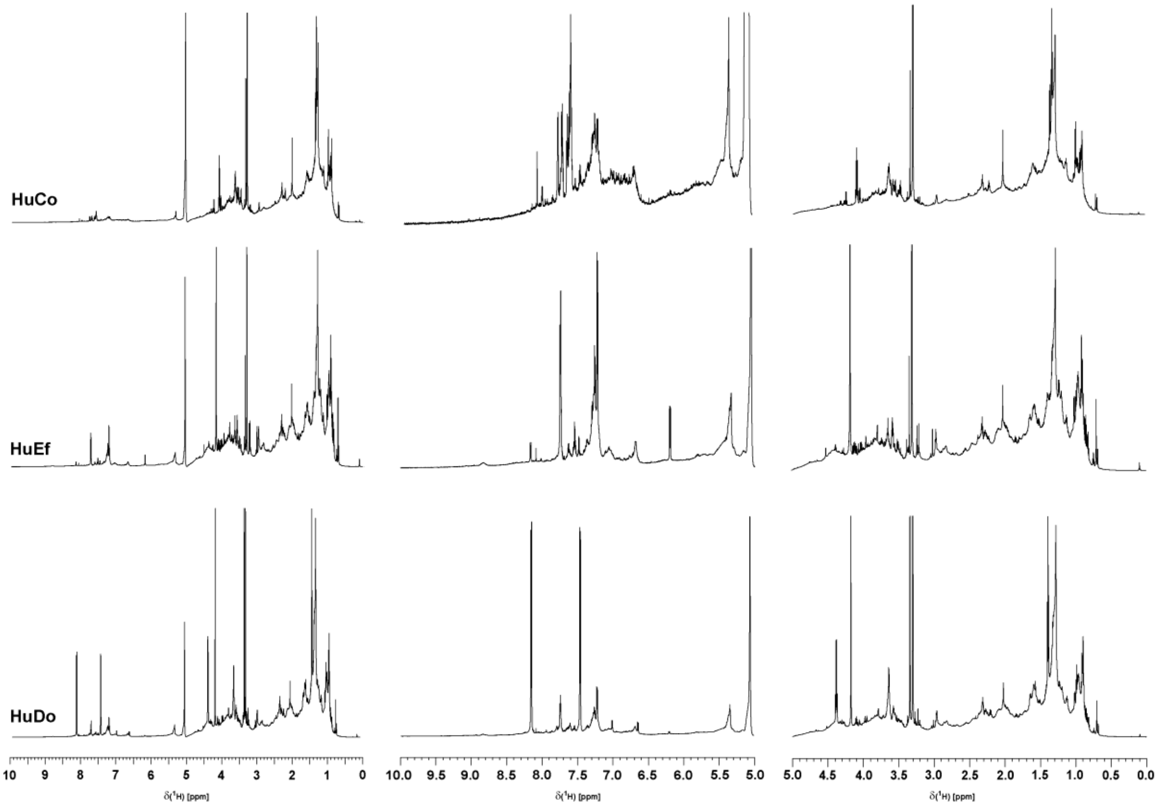

Fig. S8. ^{1}H NMR spectra (800 MHz, CD_3OD) of Huilico riverine DOM; top: pristine, control (HuCo), center: effluent (HuEf), bottom: downstream (HuDo).

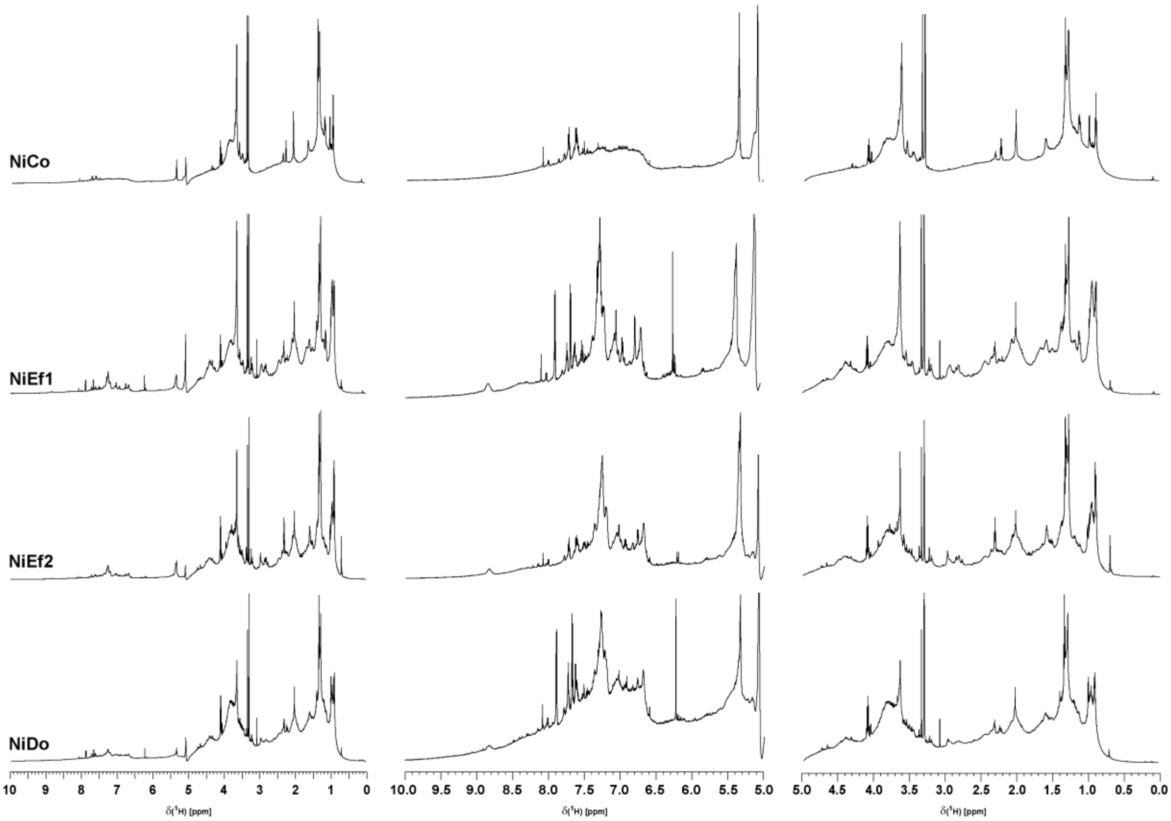

Fig. S9. ^{1}H NMR spectra (800 MHz, CD_3OD) of Niltre riverine DOM; top: pristine, control (NiCo), center: first effluent (NiEf1), second effluent (NiEf2), bottom: downstream (NiDo).

Table S3. ^{1}H NMR section integrals of pristine DOM (for a more extended version, see Table S6).

δ(^{1}H) [ppm]	key substructures	MoCo	PeCo	HuCo	NiCo
**10 - 7 ppm**	$C_{ar}\underline{\mathbf{H}}$	2.0	3.2	3.1	4.3
**7 - 5 ppm**	=C$\underline{H}$, $O_2C\underline{H}$	2.1	3.2	3.7	4.3
**5 - 3.1 ppm**	OC$\underline{\mathbf{H}}$	24.7	20.5	22.1	31.9
**3.1 - 1.9 ppm**	XCC**H;** (X: O, N)	18.7	25.1	21.9	19.3
**1.9 - 0.5 ppm**	XCCC**H;** (X: O, N)	52.5	47.9	49.3	40.2

Table S4. ^{1}H NMR section integrals of riverine DOM from all four catchments (for a more extended version, see Tables S6).

	MoCo	MoEf	MoDo	PeCo	PeEf	PeDo	HuCo	HuEf	HuDo	NiCo	NiEf1	NiEf2	NiDo
**10 - 7 ppm**	2.0	6.1	4.2	3.2	18.4	13.3	3.1	4.0	5.6	4.3	4.9	4.1	5.1
**7 - 5 ppm**	2.1	3.7	3.6	3.2	2.7	3.1	3.7	3.2	3.0	4.3	4.7	4.2	5.2
**5 - 3.1 ppm**	24.7	27.3	25.2	20.5	21.4	22.8	22.1	25.3	23.9	31.9	31.5	31.7	32.3
**3.1 - 1.9 ppm**	18.7	23.7	21.8	25.1	28.7	26.5	21.9	22.5	20.7	19.3	22.7	21.5	21.0
**1.9 - 0.5 ppm**	52.5	39.2	45.2	47.9	28.8	34.3	49.3	44.9	46.8	40.2	36.3	38.4	36.4

Table S5. ^{1}H NMR section integrals of riverine DOM from all four catchments; extended version with annotation [24].

	MoCo	MoEf	MoDo	PeCo	PeEf	PeDo	HuCo	HuEf	HuDo	NiCo	NiEf1	NiEf2	NiDo
**10.00 - 8.00 ppm**	0.3	0.8	0.9	0.8	0.8	1.0	0.5	0.7	1.6	1.0	1.2	0.9	1.4
**8.00 - 7.30 ppm**	1.0	1.9	1.9	1.7	16.0	10.7	1.6	1.8	2.6	2.2	2.0	1.8	2.3
**7.30 - 7.00 ppm**	0.6	3.4	1.4	0.7	1.6	1.6	0.9	1.6	1.4	1.1	1.7	1.5	1.4
**7.00 - 6.50 ppm**	0.6	1.0	1.0	0.8	0.8	1.0	1.0	0.7	0.8	1.5	1.4	1.3	1.7
**6.50 - 6.00 ppm**	0.2	0.3	0.6	0.7	0.3	0.5	0.7	0.6	0.4	0.7	0.9	0.7	1.1
**6.00 - 5.20 ppm**	1.3	2.3	2.0	1.7	1.5	1.6	2.0	2.0	1.7	2.0	2.4	2.2	2.4
**4.95 - 3.10 ppm**	24.7	27.3	25.2	20.5	21.4	22.8	22.1	25.3	23.9	31.9	31.5	31.7	32.3
**3.10 - 2.10 ppm**	13.8	16.5	16.0	19.9	24.1	21.6	16.6	16.6	15.3	14.8	16.5	15.2	15.6
**2.10 - 1.90 ppm**	4.9	7.2	5.7	5.2	4.7	4.9	5.3	6.0	5.4	4.5	6.2	6.2	5.4
**1.90 - 1.35 ppm**	17.3	13.8	17.7	20.7	11.5	14.3	19.9	17.7	19.0	14.1	14.6	14.7	14.6
**1.35 - 1.25 ppm**	17.6	9.5	10.2	9.3	5.3	6.7	10.7	8.2	10.3	9.0	6.2	8.1	6.5
**1.20 - 0.50 ppm**	17.6	15.8	17.2	17.9	12.0	13.4	18.6	18.9	17.5	17.2	15.4	15.6	15.3

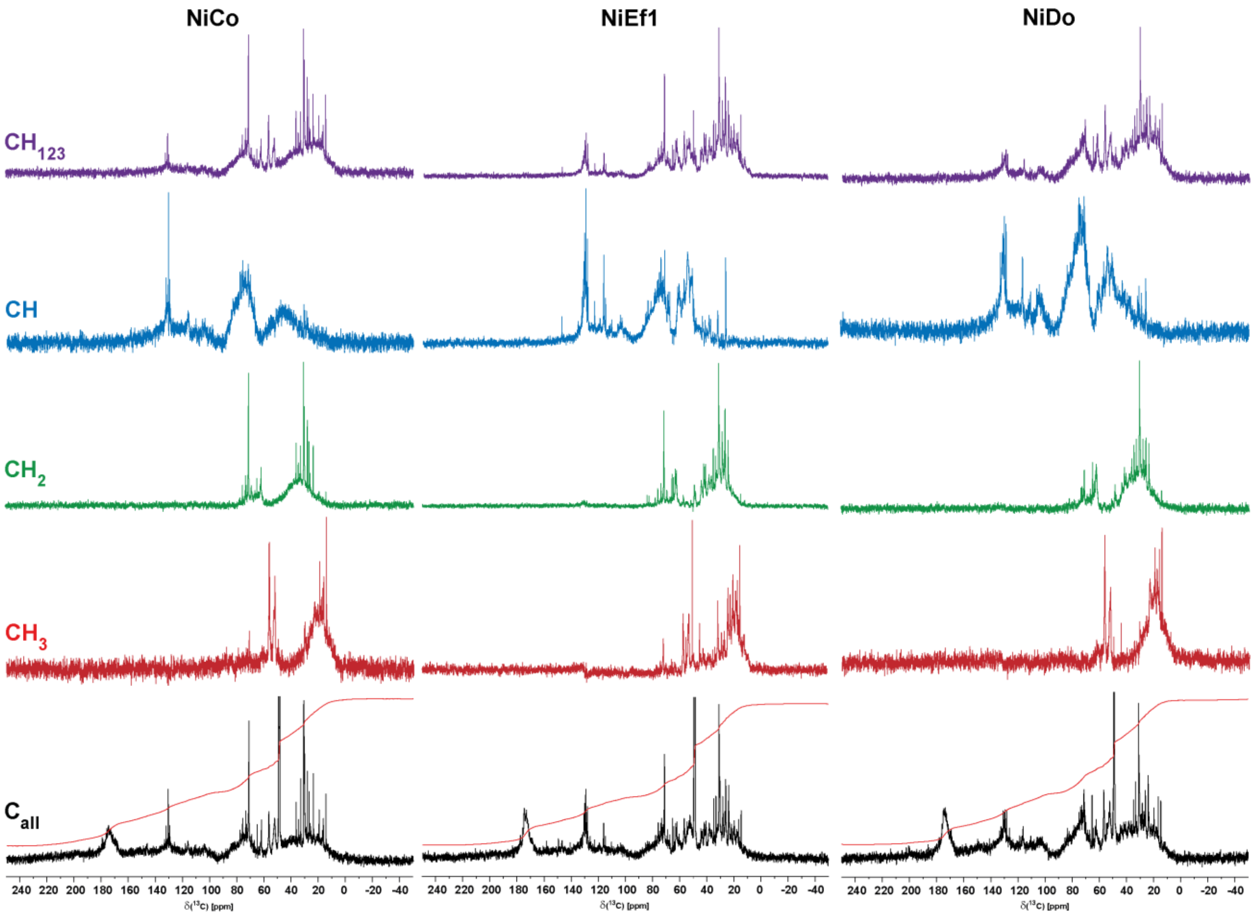

Fig. S10. Multiplicity edited ^{13}C NMR spectra of Niltre River DOM ($^{12}CD_3OD$). Left: **NiCo** SPE-DOM, center: **NiEf1** SPE-DOM, right: **NiDo** SPE-DOM. Color code: purple: CH_{123}; blue: CH; green: CH_2, red: CH_3; black: all carbon, provided with integral (cf. Table S7).

Table S6. ^{13}C NMR section integrals of Niltre DOM; H/C and O/C elemental ratios were computed from a simple reverse-mixing model[25] and showed the commonly observed displacement from FTMS-derived values, primarily owing to the ionization selectivity which applies the complex mixture of SPE-DOM molecules[26].

**$\delta(^{13}C)$ ppm**	**220-187**	**187-167**	**167-145**	**145-108**	**108-90**	**90-47**	**47-0**	**H/C ratio**	**O/C ratio**
**Key substructures**	C=O	COX	C_{ar}-O	C_{ar}-C,H	O_2CH	OCH	CCH		
**NiCo**	5.3	11.5	6.6	15.2	4.1	25.4	31.9	**1.20**	**0.69**
**NiEf1**	2.5	10.1	5.0	13.5	2.8	37.8	28.3	**1.21**	**0.71**
**NiDo**	2.4	10.3	5.5	16.6	5.5	28.1	31.6	**1.24**	**0.68**
**NMR mixing model**	**C=O**	**COOH**	**C_{ar}-O**	**C_{ar}-H**	**O_2CH**	**OCH**	**CH_2**		
H/C ratio	0	1	0	1	1	1	2		
O/C ratio	1	2	1	0	2	1	0		

**DOM (depth)**	**CH total**	**CH_2 total**	**CH_3 total**	**ratio (d_1 / c_1 / b_1 / a_1) HC_{ar}-C / O-HC-O / HC-O / HC-C**	**ratio (b_2 / a_2) H_2C-O / H_2C-C**	**ratio (b_3 / a_3) H_3C-O / H_3C-C**
**NiCo**	34	37	29	24.5 / 4.9 / 37.7 / 32.8	25.6 / 74.4	23.4 / 76.6
**NiEf1**	42	40	18	26.4 / 6.3 / 31.7 / 35.6	19.3 / 80.7	11.1 / 88.9
**NiDo**	35	48	17	24.8 / 5.7 / 37.6 / 31.9	19.7 / 80.3	16.8 / 83.2

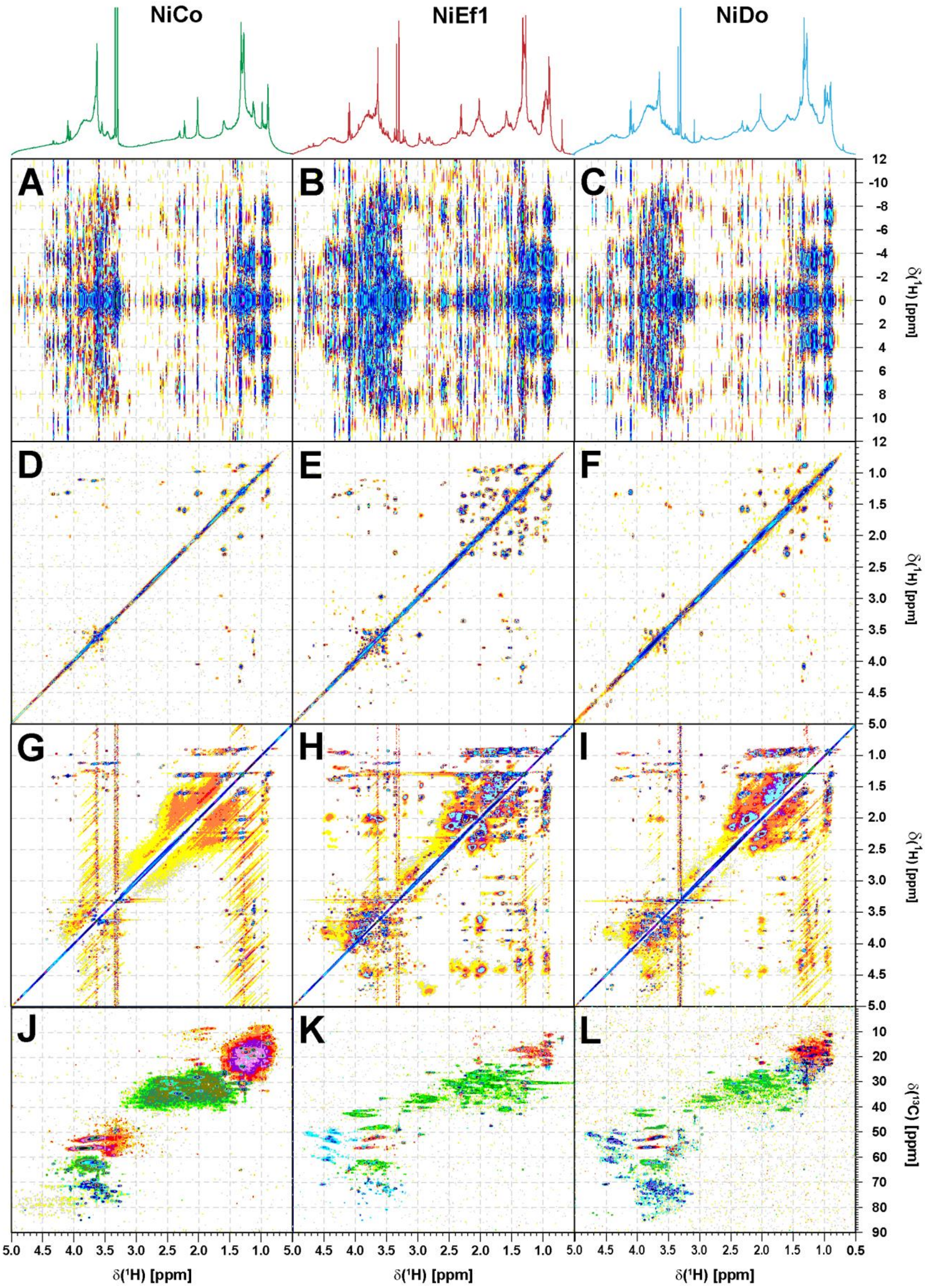

Fig. S11. Homo- (A-I) and heteronuclear (J-L) nuclear 2D NMR spectra of Niltre (left) pristine, (center) effluent and (right) downstream DOM (800 MHz, $^{12}CD_3OD$). (A-C) ^1H, ^1H JRES, (D-F) ^1H, ^1H COSY, (G-I) 1^1H, ^1H TOCSY and (J-L) ^1H, ^{13}C DEPT HSQC NMR spectra (color code: purple: CH_{123}; blue: CH; green: CH_2, red: CH_3). COSY (panel E), TOCSY (panel H) and DEPT HSQC NMR spectra (panel K) of effluent DOM are annotated in Fig. 5.

Supplementary methods

Sampling sites

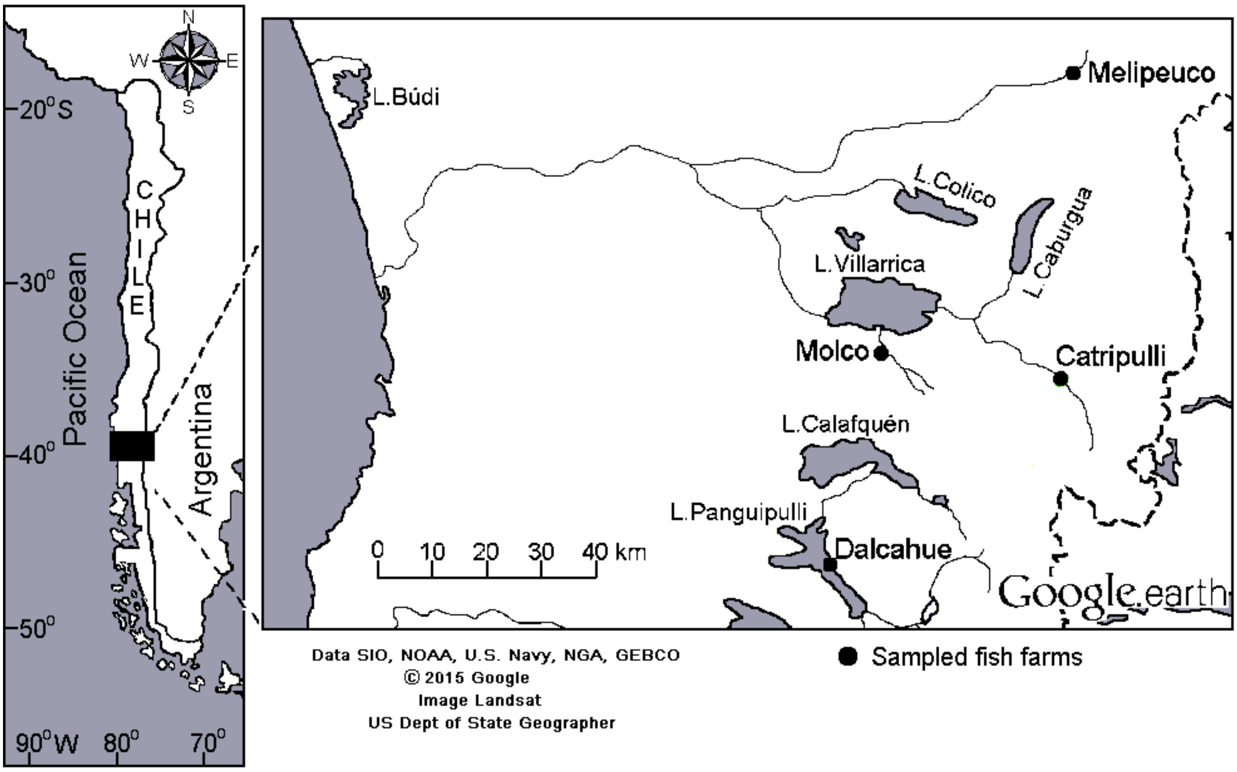

Fig. S12. Location of sampling sites in Chile. Map was adapted on basis of **Google earth** image (https://www.google.de/maps/@-39.3162606,-72.4943204,119368m/data=!3m1!1e3). The corresponding image was supplied by Data SIO, NOAA, U.S. Navy, NGA, GEBCO ©2015 Google, Image Landsat, US Dept. of State Geographer. The map was drawn using software PHOTOIMPACT version 4.2 under licence number RI630-903-61120 (https://archive.org/details/Ulead_PhotoImpact_4.2_-_Windows95-NT_UleadENG-FRE-DE).

Table S7. Acquisition conditions for NMR spectra provided in Figures. PK: NMR probehead used, 8Q: 800 MHz 5 mm cryogenic inverse quaternary $^{1}H/^{13}C/^{15}N/^{31}P$; 5D: 500 MHz 5 mm cryogenic dual $^{13}C/^{1}H$; NS: number of scans (for 2D NMR: F2); AQ: acquisition time [ms]; D1: relaxation delay [ms]; NE: number of F1 increments in 2D NMR spectra; WDW1, WDW2: apodization functions in F1 / F2 (EM exponential line broadening factor [Hz]; SI: sine bell); PR1, PR2: coefficients used for windowing functions WDW1, WDW2, EM is given in [Hz], SI derived functions indicate shift by π/n. Total NMR acquisition time AQ_{Σ} is computed as follows: $AQ_{\Sigma} = NS \times (D1 + AQ) \times NE$, with NE = 1 for 1D NMR spectra. Asterisk (*): ^{13}C DEPT NMR spectra were co-added from several individual NMR spectra.

figure	PK	NS	AQ [ms]	D1 [ms]	NE	WDW1	WDW2	PR1	PR2	amount
^{1}H	8Q	480 - 15616	5000	5000	/	/	EM	-	1	10 µg – 1 mg
JRES	8Q	2048	1000	500	64	QS	QS	0	0	1 mg
COSY	8Q	160	1000	500	1375	QS	EM	0	2.5	1 mg
TOCSY	8Q	160	1000	500	2243	QS	EM	6	2.5	150 µg
DEPT-HSQC	8Q	2048	250	1250	317	QS	EM	2.5	2.5	1 mg
HMBC	8Q	1600	1000	500	226	QS	EM	2.5	2.5	3 mg
^{13}C DEPT45	5D	496895 *	1000	2000	/	/	EM	/	1 / 25	3 mg
^{13}C DEPT90	5D	281208 *	1000	2000	/	/	EM	/	1 / 25	3 mg
^{13}C DEPT135	5D	335783 *	1000	2000	/	/	EM	/	1 / 25	3 mg
^{13}C	5D	29440	1000	19000	/	/	EM	/	1 / 25	3 mg

Supplementary references

1. Murphy, K. R., Bro, R. & Stedmon, C. A. Chemometric Analysis of Organic Matter Fluorescence. In: Paula G. Coble et al. (eds.) *Aquatic Organic Matter Fluorescence*. Cambridge Environmental Chemistry Series. Cambridge University Press. 339-375 pp. (2014).
2. Nimptsch, J. *et al.* Tracing dissolved organic matter (DOM) from land-based aquaculture systems in North Patagonian streams. *Sci. Total Environ.* **537**, 129-138 (2015).
3. Cawley, K. M. *et al.* Identifying fluorescent pulp mill effluent in the Gulf of Maine and its watershed. *Mar. Pollut. Bull.* **64.** 1678-1687 (2012b).
4. Stedmon, C. A. & Markager, S. Resolving the variability in dissolved organic matter fluorescence in a temperate estuary and its catchment using PARAFAC analysis. *Limnol. Oceanogr.* **50**, 686-697 (2005).
5. Yu, H. *et al.* Impact of dataset diversity on accuracy and sensitivity of parallel factor analysis model of dissolved organic matter fluorescence excitation-emission matrix. *Sci. Rep.* **5**, 10207 (2015).
6. Murphy, K. R. et al. Organic Matter Fluorescence in Municipal Water Recycling Schemes: Toward a Unified PARAFAC Model. *Environ. Sci. Technol.* **45**: 2909-2916 (2011).
7. Yamashita, Y., Boyer, J. N. & Jaffe, R. Evaluating the distribution of terrestrial dissolved organic matter in a complex coastal ecosystem using fluorescence spectroscopy. *Cont. Shelf Res.* **66**, 136-144 (2013).
8. Yamashita, Y., Kloeppel, B. D., Knoepp, J., Zausen, G. L. & Jaffe, R. Effects of Watershed History on Dissolved Organic Matter Characteristics in Headwater Streams. *Ecosystems* **14**, 1110-1122 (2011b).
9. Graeber, D., Gelbrecht, J., Pusch, M. T., Anlanger, C. & von Schiller, D. Agriculture has changed the amount and composition of dissolved organic matter in Central European headwater streams. *Sci. Total Environ.* **438**, 435-446 (2012).
10. Cawley, K. M., Ding, Y., Fourqurean, J. & Jaffe, R. Characterising the sources and fate of dissolved organic matter in Shark Bay, Australia: a preliminary study using optical properties and stable carbon isotopes. *Mar. Freshwater Res.* **63**: 1098-1107 (2012a).
11. Yamashita, Y., Panton, A., Mahaffey, C. & Jaffe, R. Assessing the spatial and temporal variability of dissolved organic matter in Liverpool Bay using excitation-emission matrix fluorescence and parallel factor analysis. *Ocean. Dynam.* **61**, 569-579 (2011a).
12. Garcia, R. D., Reissig, M., Queimalinos, C. P., Garcia, P. E. & Dieguez, M. C. Climate-driven terrestrial inputs in ultraoligotrophic mountain streams of Andean Patagonia revealed through chromophoric and fluorescent dissolved organic matter. *Sci. Total Environ.* **521**, 280-292 (2015).
13. Osburn, C. L. & Stedmon, C. A. Linking the chemical and optical properties of dissolved organic matter in the Baltic-North Sea transition zone to differentiate three allochthonous inputs. *Mar. Chem.* **126**, 281-294 (2011).
14. Shutova, Y., Baker, A., Bridgeman, J. & Henderson, R. K. Spectroscopic characterisation of dissolved organic matter changes in drinking water treatment: From PARAFAC analysis to online monitoring wavelengths. *Wat. Res.* **54**: 159-169 (2014).
15. Yamashita, Y., Maie, N., Briceño, H. & Jaffé, R. Optical characterization of dissolved organic matter in tropical rivers of the Guayana Shield, Venezuela. *J. Geophys. Res. Biogeosci.* **115**, 15-G00F10 (2010).
16. Brym, A. *et al.* Optical and chemical characterization of base-extracted particulate organic matter in coastal marine environments. *Mar. Chem.* **162**: 96-113 (2014).
17. Tanaka, K., Kuma, K., Hamasaki, K. & Yamashita, Y. Accumulation of humic-like fluorescent dissolved organic matter in the Japan Sea. *Sci. Rep.* **4**, 1-7 (2014).
18. Kowalczuk, P., Tilstone, G. H., Zablocka, M., Roettgers, R. & Thomas, R. Composition of dissolved organic matter along an Atlantic Meridional Transect from fluorescence spectroscopy and Parallel Factor Analysis. *Mar. Chem.* **157**, 170-184 (2013).
19. Stedmon, C. A. *et al.* Characteristics of dissolved organic matter in Baltic coastal sea ice: allochthonous or autochthonous origins? *Environ. Sci. Technol.* **41**, 7273-7279 (2007a).
20. Stedmon, C. A. *et al.* Photochemical production of ammonium and transformation of dissolved organic matter in the Baltic Sea. *Mar. Chem.* **104**, 227-240 (2007b).
21. Murphy, K. R., Ruiz, G. M., Dunsmuir, W. T. M. & Waite, T. D. Optimized parameters for fluorescence-based verification of ballast water exchange by ships. *Environ. Sci. Technol.* **40**, 2357-2362 (2006).
22. Murphy, K. R., Stedmon, C. A., Waite, T. D. & Ruiz, G. M. Distinguishing between terrestrial and autochthonous organic matter sources in marine environments using fluorescence spectroscopy. *Mar. Chem.* **108**, 40-58 (2008).
23. Herzsprung, P. *et al.* Variations of DOM quality in inflows of a drinking water reservoir: Linking of van Krevelen diagrams with EEMF spectra by rank correlation. *Environ. Sci. Technol.* **46**: 5511-5518 (2012).
24. Dvorski, S. E.-M. *et al.* Geochemistry of dissolved organic matter in a spatially highly resolved groundwater petroleum hydrocarbon plume cross-section. *Environ. Sci. Technol.* **50**, 5536–5546 (2016).
25. Hertkorn, N., Harir, M., Koch, B. P., Michalke, B. & Schmitt-Kopplin, P. High-field NMR spectroscopy and FTICR mass spectrometry: powerful discovery tools for the molecular level characterization of marine dissolved organic matter. *Biogeosciences* **10**, 1583-1624 (2013).

26. Hertkorn, N. *et al.* Natural organic matter and the event horizon of mass spectrometry. *Anal. Chem.* **80**, 8908-8919 (2008).
